# Hybrid RBF neural network–based sliding mode control for a magnetorheological elastomer isolator under variable frequency and amplitude excitations

**DOI:** 10.1371/journal.pone.0360069

**Published:** 2026-10-09

**Authors:** Kiran Katari, Umanath R. Poojary, Ananda Hegde, Gangadharan K. V.

**Affiliations:** 1 Department of Mechanical Engineering, National Institute of Technology, Mangalore, Karnataka, India; 2 Department of Mechanical and Manufacturing Engineering, Manipal Institute of Technology, Udupi, Karnataka, India; National University of Sciences and Technology, PAKISTAN

## Abstract

Magnetorheological elastomers have attracted significant attention for semi-active vibration isolation applications due to their ability to rapidly change mechanical properties under an applied magnetic field. However, the nonlinear and hysteretic behavior of Magnetorheological elastomer materials poses challenges for achieving robust vibration suppression under varying excitation conditions. This study presents a hybrid control strategy that integrates a radial basis function neural network with a Sliding Mode Controller to enhance the vibration isolation performance of an MRE-based semi-active isolator. A MRE isolator with orthogonal magnetic flux configuration is designed and fabricated, and its dynamic characteristics are evaluated under harmonic excitation in the frequency range of 15–80 Hz. The proposed hybrid controller adaptively estimates system nonlinearities using the neural network while maintaining robustness through sliding mode control. Experimental and simulation results demonstrate that the proposed controller provides effective vibration attenuation while maintaining stable adaptive performance under varying excitation frequency and amplitude. The results show that the semi-active MRE isolator achieves a maximum vibration reduction of 55.8% near the resonance region under the investigated operating conditions, while maintaining stable performance across varying excitation frequencies. The proposed control framework provides an effective solution for handling nonlinear dynamics, and it offers greater adaptability in smart vibration isolation systems based on magnetorheological elastomers.

## 1 Introduction

Magnetorheological elastomers (MREs) are a class of smart composite materials consisting of ferromagnetic particles embedded in an elastomeric matrix. When subjected to an external magnetic field, the mechanical properties of MREs, such as stiffness and damping, can be rapidly and reversibly tuned. This field-dependent behavior makes MREs highly attractive for applications in vibration isolation, adaptive structures, and smart mechanical systems. Compared with magnetorheological fluids, MREs offer advantages such as structural stability, absence of particle sedimentation, and ease of integration into mechanical components. Consequently, MRE-based devices have been extensively investigated for semi-active vibration control applications in recent years [1–4].

Significant research efforts have been devoted to improving the performance of MRE materials through particle distribution, matrix composition, and fabrication techniques. Early studies demonstrated that the mechanical properties of MREs strongly depend on particle concentration, particle alignment, and the type of elastomer matrix used during fabrication [5–7]. Isotropic and anisotropic MRE structures have been widely investigated, where anisotropic MREs typically exhibit higher magneto-induced stiffness due to the chain-like alignment of magnetic particles during curing under magnetic fields [8–10].

Recent studies have further explored the effects of hybrid particle compositions, microstructural optimization, and advanced fabrication techniques to enhance the magneto-mechanical properties of MREs. Researchers have reported significant improvements in magnetorheological effects through the incorporation of nano-scale additives and optimized curing processes [11–14]. Moreover, advances in material characterization techniques have enabled better understanding of the nonlinear viscoelastic behavior and magnetic field-dependent stiffness variation in MRE materials [15–18].

Along with material development, numerous MRE-based vibration mitigation devices have been proposed and experimentally validated. These include MRE vibration isolators, adaptive mounts, sandwich beams, and tuned vibration absorbers designed to mitigate unwanted vibrations in mechanical and structural systems. The field-dependent stiffness of MRE materials allows such devices to adapt their dynamic properties according to changing excitation conditions [19–21].

Recent studies have demonstrated the feasibility of MRE isolators in applications such as vehicle suspension systems, structural vibration mitigation, and precision engineering platforms. Researchers have also developed multi-layer and shear-mode MRE devices capable of providing enhanced vibration suppression performance over wider frequency ranges [22–25]. These developments highlight the potential of MRE-based devices as effective semi-active vibration isolation solutions.

By virtue of elastomer matrix, MRE is a viscoelastic material, additionally it attains attributes of a polymer composite due to the ferromagnetic fillers. Under the influence of magnetic field, MRE exhibits field dependent properties. These factors make MRE a complex engineering material which requires integration of the aforementioned domains when modeling the material behavior. Due to the highly nonlinear and hysteretic behavior of MRE materials, accurate modeling plays a crucial role in the design and control of MRE-based vibration isolation systems. Several phenomenological and physics-based models have been proposed to describe the dynamic behavior of MRE materials under varying magnetic fields. Commonly used models include viscoelastic models, Bouc–Wen hysteresis models, and modified Kelvin–Voigt models [26].

Among these approaches, the Bouc–Wen model has been widely adopted due to its ability to capture nonlinear hysteresis behavior across different excitation conditions. Researchers have proposed modified and improved Bouc–Wen models capable of representing the dynamic characteristics of MRE materials over wide frequency ranges and strain amplitudes [27,28]. In addition, data-driven modeling approaches and machine learning techniques have recently been explored to improve modeling accuracy for complex smart material systems [29].

Apart from material model, design and development of MRE based devices require an effective control strategy that can make the system adoptable. A considerable research effort has focused on the development of control strategies for MRE-based vibration isolation systems. Traditional model-based and model-free controllers such as proportional–integral–derivative (PID) controllers have been widely used due to their simplicity and ease of implementation in semi-active vibration control systems [30].

To address nonlinear system behavior and modeling uncertainties, researchers have proposed intelligent control approaches such as fuzzy logic controllers and neural network-based controllers. These techniques can effectively handle nonlinearities without requiring precise mathematical models of the system [31]. Neural network-based controllers, in particular, have demonstrated strong capability in learning complex nonlinear system dynamics and improving vibration suppression performance in smart structures [32]. More advanced control techniques such as sliding mode control (SMC) have attracted significant attention due to their robustness against parameter uncertainties and external disturbances. Adaptive control strategies have also been developed to automatically adjust controller parameters in response to varying operating conditions [33]. Furthermore, optimization-based algorithms and hybrid intelligent control frameworks combining fuzzy logic, neural networks [34], and robust nonlinear control techniques [32,35–37] have been proposed to further enhance vibration mitigation performance in smart vibration isolation systems.

In recent years, intelligent control techniques for smart vibration isolation systems have evolved considerably. Recent studies have increasingly adopted adaptive neural networks, adaptive fuzzy sliding mode control, and hybrid optimization-based approaches to improve the performance of smart dampers and magnetorheological elastomer (MRE) isolators operating under nonlinear and uncertain conditions [31–33]. These intelligent controllers enhance adaptability by learning unknown system dynamics online while simultaneously reducing control chattering and improving robustness against parameter uncertainties. Furthermore, hybrid control architectures integrating artificial intelligence with robust nonlinear control techniques have demonstrated superior vibration attenuation, faster convergence, improved disturbance rejection, and enhanced energy efficiency in semi-active smart vibration isolation systems [35–38]. These recent developments further motivate the present study, where a Hybrid Radial Basis Function Neural Network–Sliding Mode Controller is proposed to exploit both the nonlinear approximation capability of neural networks and the robustness of sliding mode control for MRE-based vibration isolation.

Despite significant advances in MRE material development, device design, modeling techniques, and control strategies, several challenges remain in achieving robust vibration suppression across a wide range of operating conditions. First, the nonlinear and hysteretic behavior of MRE materials introduces uncertainties that complicate the modeling and control of vibration isolation systems. Second, many existing vibration isolation devices are limited to specific deformation modes, which restricts their ability to effectively attenuate multi-directional vibrations. Third, conventional control strategies may not provide sufficient adaptability when the system is subjected to varying excitation conditions. To address these challenges, hybrid intelligent control strategies that integrate learning-based algorithms with robust nonlinear control methods have recently attracted increasing attention. Such approaches can improve system adaptability while maintaining stability under uncertain dynamic environments. The scientific contribution of the present work extends beyond the implementation of an intelligent controller. Unlike conventional Sliding Mode Control, which relies heavily on an accurate mathematical representation of the system dynamics, the proposed hybrid framework integrates a Radial Basis Function Neural Network (RBFNN) with a physics-based Bouc–Wen hysteresis model to adaptively compensate for nonlinear modeling uncertainties associated with the hysteretic behavior of the MRE isolator. The Bouc–Wen model preserves the physical interpretation of the hysteresis mechanism, while the RBFNN estimates the residual nonlinear dynamics arising from parameter variations, excitation-dependent characteristics, and unmodeled disturbances. Consequently, the proposed framework combines the robustness of Sliding Mode Control with the adaptive learning capability of the RBFNN, resulting in improved vibration suppression under simultaneously varying excitation frequency and amplitude without requiring an exact nonlinear system model. Therefore, there is a strong motivation to develop advanced hybrid intelligent control strategies capable of handling nonlinear hysteretic dynamics while maintaining robust vibration isolation performance over varying excitation frequencies and amplitudes. To address this challenge, this study proposes a Hybrid Radial Basis Function Neural Network–Sliding Mode Controller (RBSM) for an MRE-based semi-active vibration isolator. The proposed framework combines a physics-based Bouc–Wen hysteresis model with adaptive neural network learning to compensate for nonlinear modeling uncertainties while preserving the robustness of Sliding Mode Control. The effectiveness of the proposed controller is evaluated under variable-frequency and variable-amplitude excitation conditions representative of practical operating environments.

## 2 MRE isolator design

In the present study, a magnetorheological elastomer (MRE) vibration isolator operating in the shear mode is designed and fabricated. The shear-mode configuration is selected because it provides relatively low initial stiffness, which allows significant stiffness modulation when a magnetic field is applied. Consequently, a larger controllable stiffness range can be achieved compared with compression-mode configurations, thereby improving vibration isolation performance.

The fabricated components and schematic representation of the proposed MRE isolator are shown in Fig 1(a) and (b). The isolator mainly consists of a permanent electromagnet assembly, a movable central core, two ring-shaped MRE elements, and a non-magnetic aluminum piston connected to the movable core [Fig 1(a)]. These components together form the main body of the vibration isolator. The cross-section of the isolator assembly is shown in Fig 1(b). Electromagnet consists of a copper coil wound using 20 AWG gauge wire with approximately 980 turns. The coil is wrapped around a cylindrical bobbin having an inner diameter of 20 mm, an outer diameter of 100 mm, and a height of 40 mm. Low-carbon steel is used as the material for both the fixed and movable magnetic cores due to its high magnetic permeability (relative permeability $\left(\mu_{r}\approx 100\right)$), which helps to enhance the magnetic field intensity and efficiently guide the magnetic flux through the active MRE region. Two ring-shaped silicone elastomer-based MRE elements are employed as the resilient components of the isolator. Each MRE element has an inner diameter of 40 mm, an outer diameter of 50 mm, and a thickness of 5 mm. These elements are positioned between the fixed and movable steel cores at the top and bottom of the isolator, allowing shear deformation when external vibrations are applied. A non-magnetic aluminum piston is connected to the movable steel core in order to transmit motion from the excitation source to the MRE elements. The use of aluminum minimizes magnetic interference while maintaining structural rigidity. The magnetic circuit is designed such that a stationary steel plate core and a centrally positioned movable cylindrical steel core concentrate the magnetic flux through the MRE elements.

**Fig 1:**
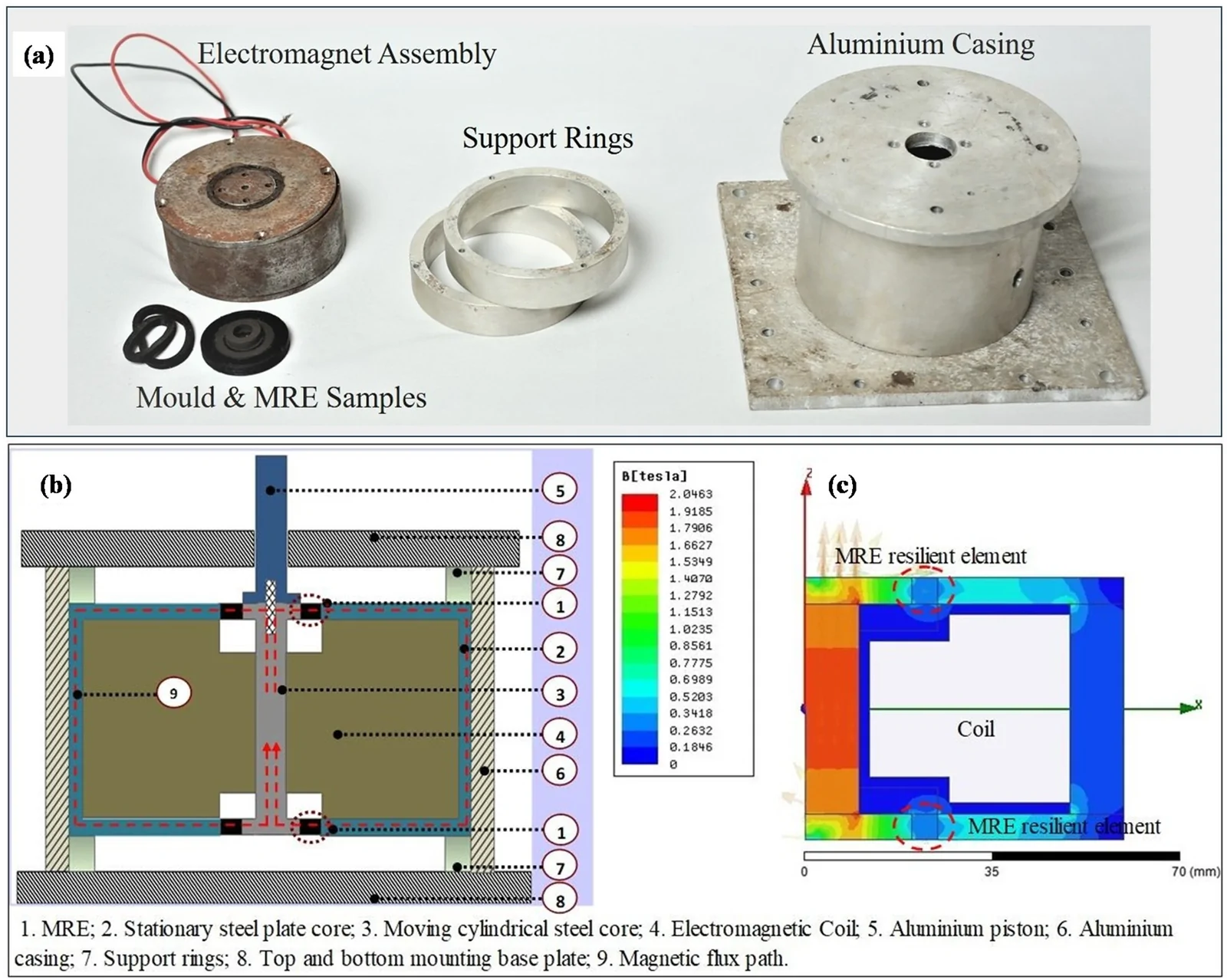
(a) The fabricated components of the isolator, (b) Schematic representation of MRE isolator and (c) magnetic field simulation of a cross-section of the isolator using ANSYS Maxwell 15.

In this configuration, the magnetic flux lines generated by the electromagnetic coil are oriented orthogonally to the loading direction of the MRE elements. This orthogonal flux configuration enhances the interaction between the magnetic field and the ferromagnetic particles embedded in the elastomer matrix, thereby improving the tunability of the effective stiffness and damping properties of the isolator. To analyze the magnetic flux path and field distribution within the isolator, magnetic field simulations were conducted using ANSYS Maxwell. The effective magnetic permeability of the MRE composite was estimated using the Maxwell–Garnett mixing rule [39]. The simulation results confirmed that the magnetic flux lines are effectively guided through the MRE elements Fig 1(c). Under an input current of 3 A supplied to the electromagnet, a magnetic flux density of approximately 0.38 T is generated within the MRE elements. The isolator assembly is enclosed within a cylindrical aluminum casing with an inner diameter of 120 mm, an outer diameter of 150 mm, and a height of 170 mm. To facilitate the smooth motion of the movable core, two aluminum support rings (inner diameter 100 mm, outer diameter 120 mm, height 25 mm) are installed at the top and bottom of the casing. Additionally, two circular mounting plates with a diameter of 180 mm and a thickness of 10 mm are provided at the top and bottom of the isolator to ensure secure installation. The lower mounting plate is used to attach the isolator assembly to the electrodynamic shaker during vibration experiments, while the upper mounting plate supports the payload mass. The proposed design offers a compact and modular structure that enables efficient magnetic flux utilization and significant tunability of the mechanical properties of the MRE elements. Furthermore, with minor structural modifications, the same configuration can be adapted for torsional vibration isolation in rotating machinery applications.

## 3 Synthesis of MRE

Previous research has demonstrated that silicone rubber, utilized as a matrix for MRE isolators, can attain a significant relative MR impact [40]. For the suggested design, two ring-shaped MRE resilient elements are fabricated with an inner diameter of 40 mm, an outer diameter of 50 mm, and a thickness of 5 mm. The MRE is manufactured using a silicone matrix and carbon iron particle (CIP) fillers. The silicone matrix is purchased from Aditya Silicone Rubber (MurtiSil, Rtv 1010), and the CIP fillers are of type CC (manufacturer: BASF, size distribution: 3–5 μm). Previous studies indicate that an optimal weight ratio of 72% CIP is selected for the synthesis procedure [40–43]. Furthermore, silicone oil (polydimethylsiloxane, 100 cSt viscosity, Sigma–Aldrich) is included as an additive to facilitate the synthesis process. The materials are meticulously combined in a weight ratio of 72% carbonyl iron, 16% silicone rubber, and 12% silicone oil, and the resultant combination is cast into the mold. The mold was placed in the vacuum chamber for roughly 20 minutes to remove any entrapped air bubbles in the mixture. Subsequent to the vacuuming procedure, the MRE samples were cured for approximately 24 hours at ambient temperature under sustained pressure [41].

## 4 Experimental methodology and experimental setup

The performance of the MRE isolator depends on the field-dependent characteristics of the MRE resilient element. The efficacy of the MRE isolator is assessed by displacement transmissibility testing. The employed methodology is depicted in Fig 2. The isolator system is modelled as a single degree of freedom (SDOF) system (Fig 2). The resilient component of the isolator is represented as the elastomeric spring. One extremity of the spring is affixed to the proof mass, while the other extremity is secured to the excitation base. A sine sweep signal (Fig 2) is applied to the base, and the resultant response (Fig 2) is measured at the moving mass. The frequency response function is defined as the ratio of the displacement transmissibility between the output and input ends, utilizing dual channel spectrum measurement. The frequency response plot is expressed as the ratio of the output response and the input excitation. A typical frequency-response plot is illustrated in Fig 2. The peak denotes the system’s natural frequency, at which the largest displacement is transmitted to the moving mass. From the peak response, the natural frequency and the damping ratio are estimated.

**Fig 2:**
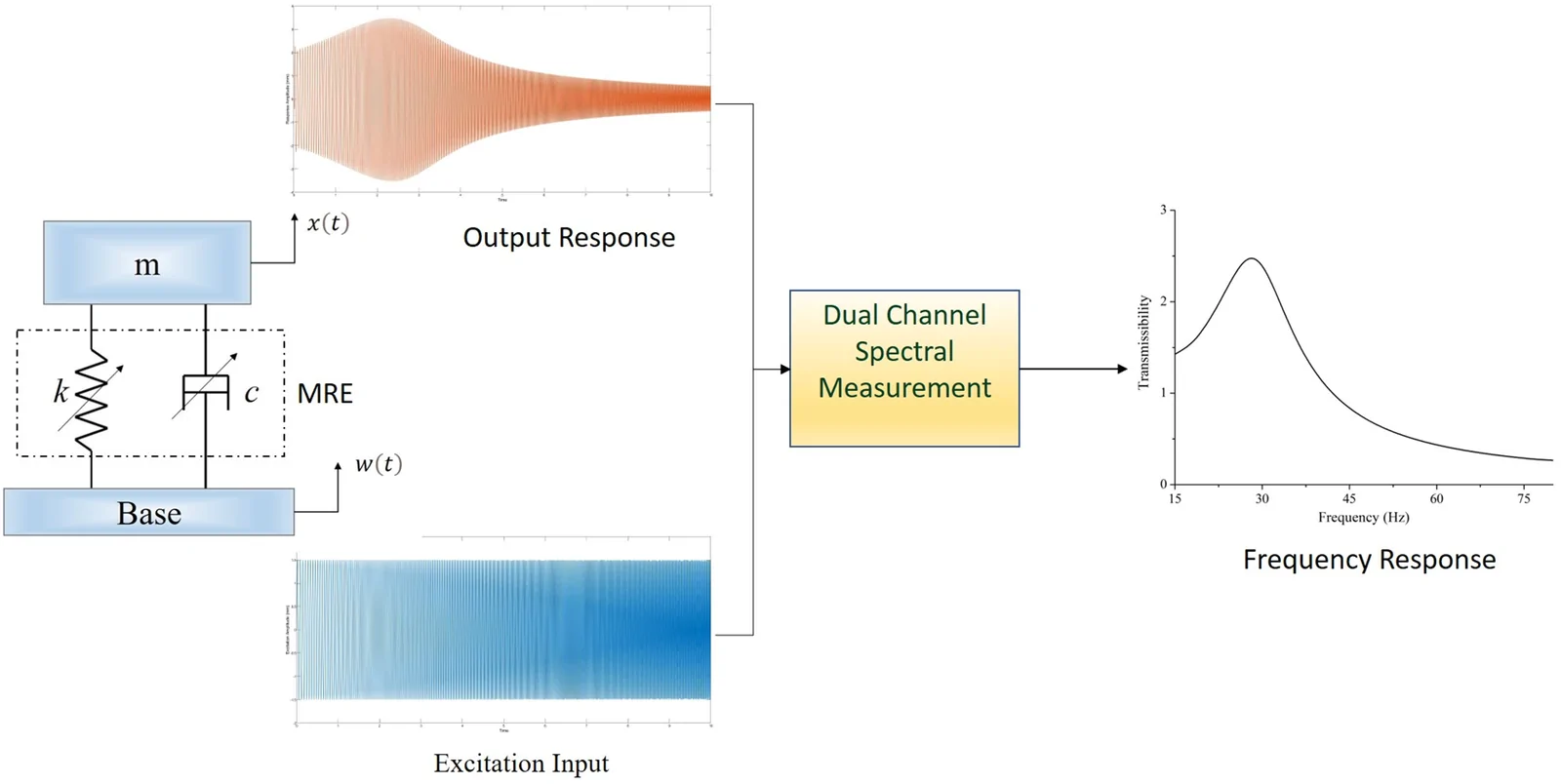
Methodology to assess the displacement transmissibility response of MRE isolator.

The experimental apparatus for measuring displacement transmissibility is illustrated in Fig 3. The MRE isolator was fastened to an inertial mass of 0.583 kg and mounted on the electrodynamic shaker (APS 420 ELECTRO-SEIS). Two KISTLER (type 8774A50) accelerometers were employed to measure the input and output acceleration; one was positioned on the base plate and the other atop the inertial mass. The MRE isolator is connected to a programmable power supply that provides an input current of up to 3 A to the coil. The preliminary evaluation of the MRE isolator in passive mode indicated a natural frequency of around 30 Hz, with a corresponding value of 50 Hz at an input current of 3 A. Therefore, to evaluate the performance of the MRE isolator, a vicinity around the resonance region is examined. The performance evaluation of the MRE isolator concentrates on the frequency range of 15 Hz to 80 Hz. The experiments are conducted at different amplitudes, excitation frequencies, and magnetic field intensities to evaluate the changes in isolation performance. To examine the impact of amplitude dependency on the performance of the MRE isolator, experiments were conducted at various excitation amplitudes ranging from 1.25 mm to 2.25 mm. Initial studies were performed to calibrate the voltage output of the function generator concerning amplitude dependency, where 2 V corresponds to an excitation amplitude of 1.25 mm, and 4 V corresponds to an excitation amplitude of 2.25 mm. A sine sweep frequency ranging from 15 Hz to 80 Hz was produced over a time of 8 seconds from the function generator to the shaker, with the disturbances transmitted to the inertial mass through the MRE isolator. For each test, the current supplied to the coil ranged from 0 to 3 A, increasing in increments of 0.5 A. Responses from the accelerometer sensor at the base and inertial mass were gathered (2.5 k samples in 8 seconds) with a data collection device (NI 9234) and processed with LabVIEW software to get the frequency response function expressed in terms of displacement transmissibility.

**Fig 3:**
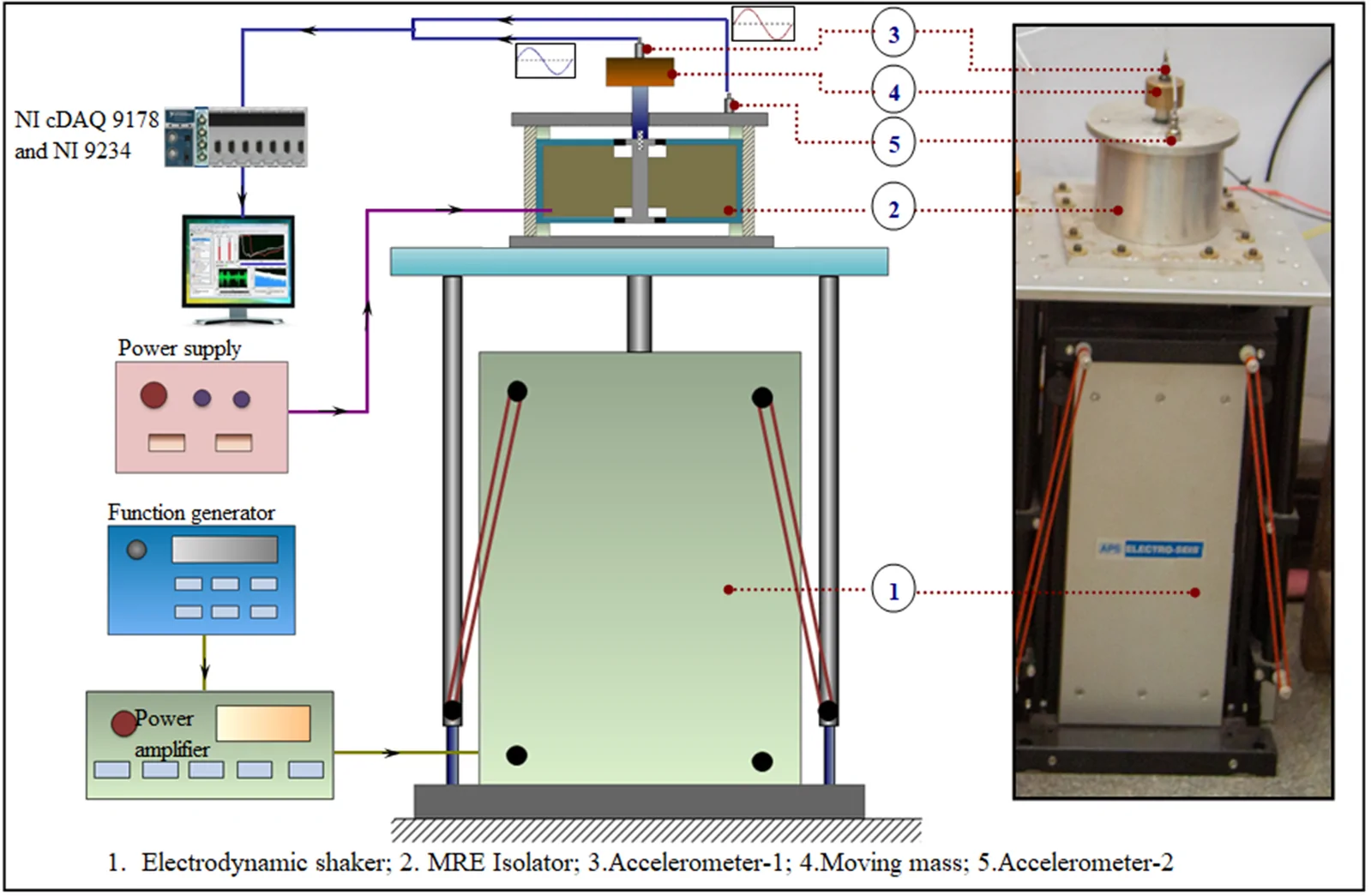
Schematic representation of the test setup for measuring the displacement transmissibility response of the isolator.

## 5 Displacement transmissibility response of the MRE isolator

The displacement transmissibility responses of the MRE isolator provide crucial insights into the ability of the isolator to mitigate the vibration. The displacement transmissibility responses of the MRE isolator at different input currents under a 1.5 mm amplitude of excitation is presented in Fig 4. Displacement transmissibility response would be maximum at the natural frequency, indicating the resonance region of the system. As evident from the plots, the frequency corresponding to the peak amplitude, which increases with the increase in input current. This indicates the increase in the stiffness of MRE resilient element under the influence of the magnetic field. For example, at 3 A input current, the natural frequency rises to 47.2 Hz, accompanied by a frequency shift of 19.2 Hz.

**Fig 4:**
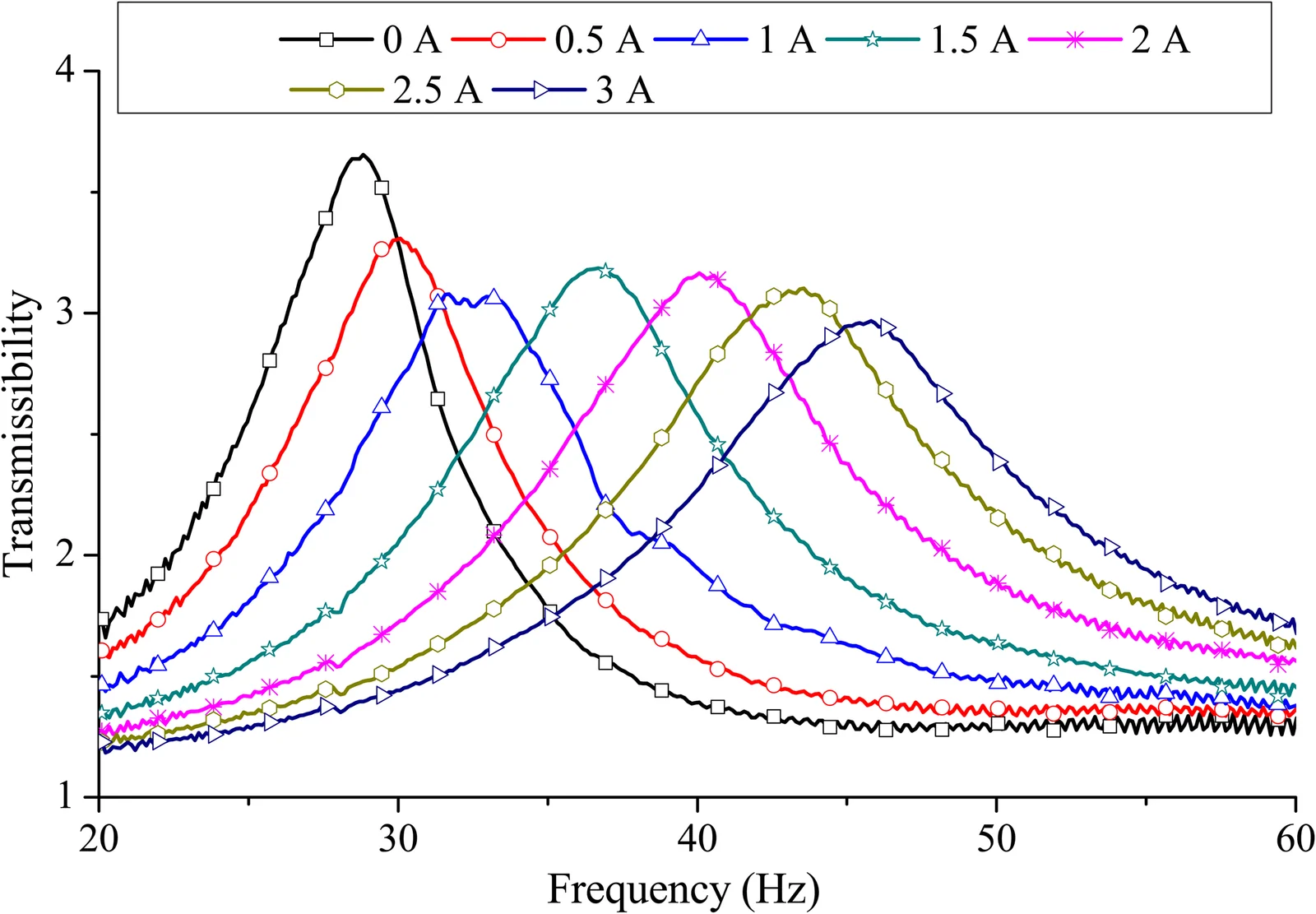
Displacement transmissibility of MRE isolator at different current input.

The values presented in Table 1 correspond to the variation in the natural frequency of the MRE isolator under different magnetic field conditions. The shift in natural frequency is primarily caused by the change in the effective stiffness of the MRE element when subjected to a magnetic field. It should be noted that the natural frequency shift reflects the stiffness variation of the isolator rather than the damping ratio. This shift in natural frequency, with the increase in input current is detailed in Table 1. These details confirm the intricate relationship between magnetic field strength and natural frequency, forming a fundamental basis for effective controller design.

**Table 1:** Natural frequency and frequency shift of the MRE isolator under various current inputs.

Current (A)	Natural frequency (Hz)	Frequency shift (Hz)
0	28.00	–
0.5	29.87	1.87
1	33.00	5.00
1.5	37.12	9.12
2	41.50	13.50
2.5	44.62	16.62
3	47.20	19.20

The response of MRE also depends on the input amplitude of displacement. Fig 5 further provides a comparison of the displacement transmissibility responses at different amplitudes of excitation for both passive (0 A) and active (3 A) states. This figure visually captures the sensitivity of the MRE isolator to varying excitation amplitudes. As the amplitude of displacement increases, the frequency corresponding to the maximum displacement transmissibility decreases. This confirms the fact that, like conventional polymer composites, the MRE resilient element also exhibits amplitude dependent behavior. This trend is consistent even under the influence of the magnetic field. For example, at an excitation amplitude of 2.25 mm and an input current of 3 A, the natural frequency decreases to 43.09 Hz, compared to 52.70 Hz at 1.25 mm excitation. This corresponds to a frequency shift from 21.07 Hz (at 1.25 mm excitation) to 16.09 Hz. The frequency shift in MRE isolator as a function of input excitation is compiled in Table 2.

**Table 2:** Properties of MRE isolator under different amplitudes of excitation.

Excitation Amplitude (mm)	Without Magnetic Field (0 A)			With Magnetic Field (3 A)			Frequency Shift (Hz)
	Natural Frequency (Hz)	Stiffness (kN/m)	Damping Ratio	Natural Frequency (Hz)	Stiffness (kN/m)	Damping Ratio	
1.25	31	22.03	0.28	52.70	63.93	0.22	21.07
1.50	28	17.98	0.17	47.19	51.09	0.18	19.18
1.75	27.6	17.47	0.14	44.59	45.61	0.18	17.00
2.00	27.3	17.09	0.10	44.09	44.60	0.17	16.79
2.25	27	16.72	0.01	43.09	42.60	0.16	16.09

**Fig 5:**
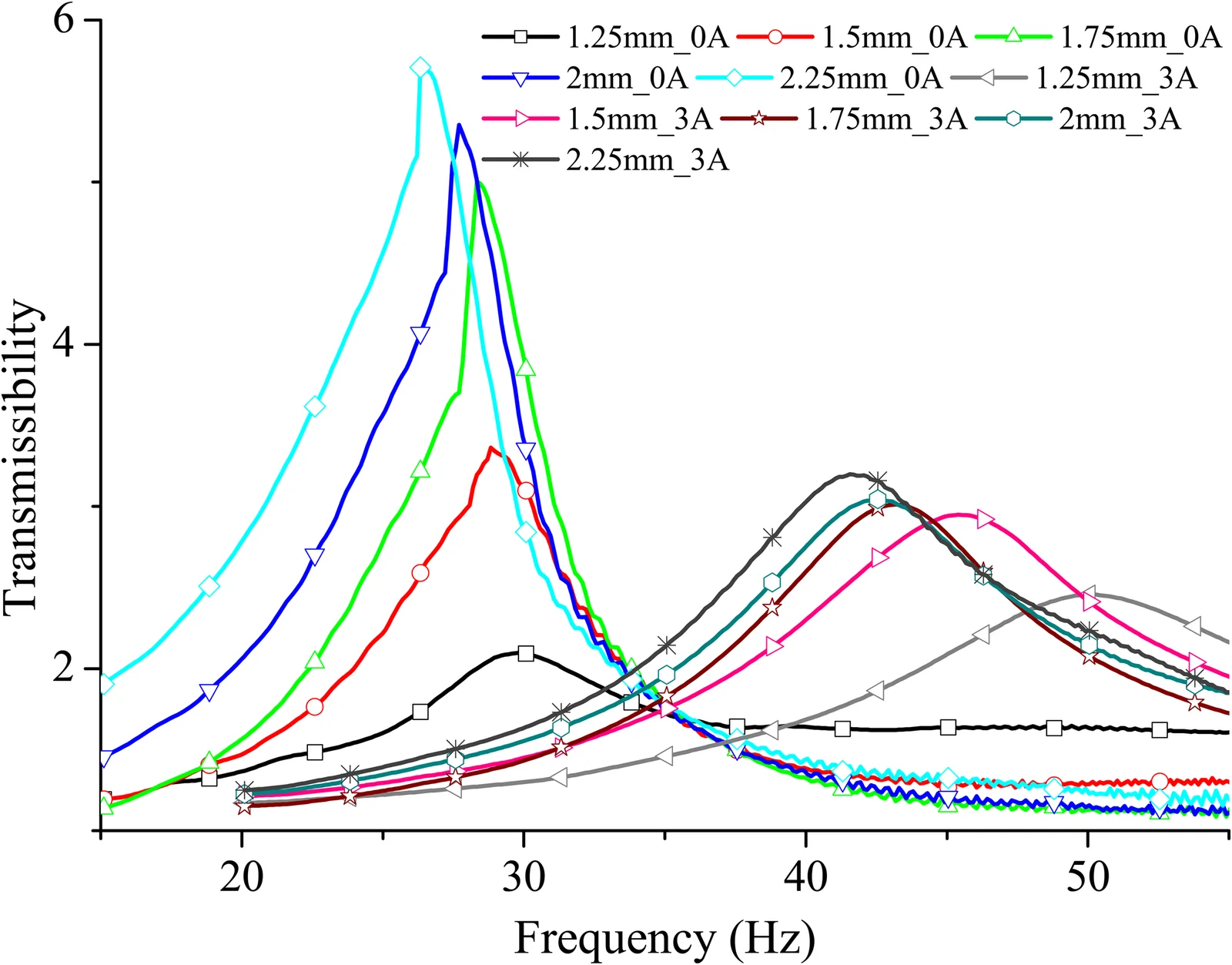
Transmissibility of MRE isolator with different excitation input without magnetic field 0 A and with magnetic field 3 A.

The amplitude dependent behavior is a typical characteristic often found in the filled elastomers. The MRE also belongs to a class of filled elastomer with additional field induced attributes. At lower excitation amplitudes, the frequency shift is relatively larger compared to the frequency shift registered at higher excitation amplitudes. The application of a magnetic field alters the mechanical properties of the MRE element, and which in turn alters the amplitude dependent variation in the characteristics of the isolator. In the passive state (without magnetic field), the stiffness of the elastomer matrix is relatively low, resulting in a lower natural frequency of the isolator. When a magnetic field is applied, the embedded ferromagnetic particles interact and form chain-like structures along the magnetic field direction. This interaction increases the effective stiffness of the MRE element and shifts the natural frequency of the isolator to a higher value. On the contrary, increased excitation amplitude modifies the interaction between the fillers, which eventually contributes to the variation in the frequency shift (from active to passive state) as a function of excitation amplitude.

Due to this stiffness variation, the resonance frequency of the isolator changes between passive and active states. If the excitation frequency is close to the passive state natural frequency, large vibration amplitudes may occur due to resonance. By activating the magnetic field, the stiffness increases and the resonance frequency shifts away from the excitation frequency, thereby reducing the vibration amplitude. However, maintaining the isolator continuously in the active state may also lead to resonance when the excitation frequency approaches the active state natural frequency. Therefore, it is necessary for the controller to switch between passive and active states depending on the excitation frequency. A typical displacement transmissibility plot illustrating this behavior is presented in Fig 6. According to this plot, the MRE isolator must be under active state to overcome the resonance of the isolator system under passive state. Continually maintaining the active state is not beneficial as it leads to the risk of encountering the resonance of the active state. Thus, a frequency needs to be defined that marks the transition from the active state to the passive state. This frequency is defined as the Controller Stopping Frequency [44].

**Fig 6:**
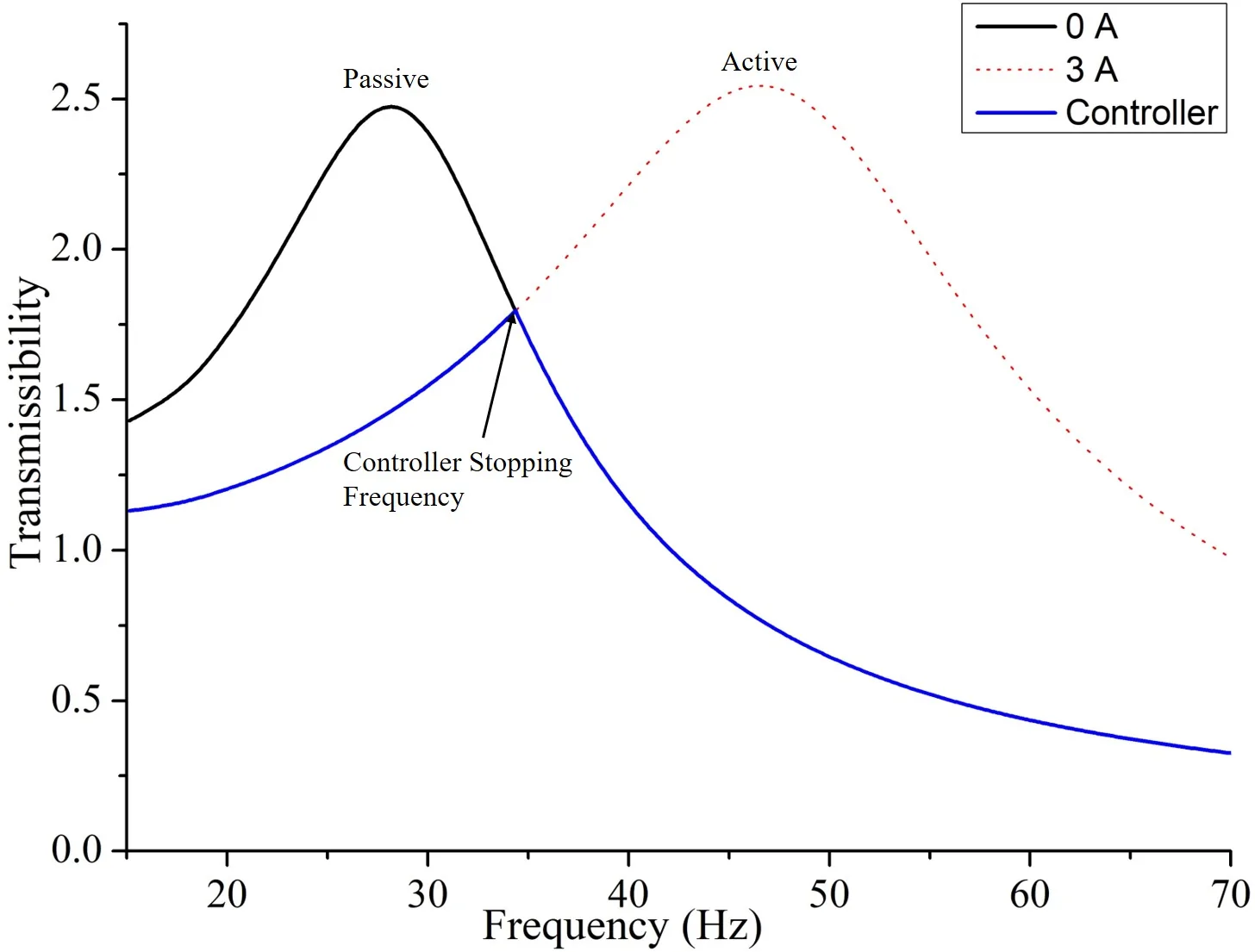
Variation of natural frequency of the MRE isolator under different magnetic field conditions.

The controller stopping frequency, $f_{s}$, is defined as the excitation frequency at which the additional benefit of active control becomes negligible compared with the passive response of the isolator. In the present study, this criterion is evaluated using the displacement transmissibility of the passive and controlled states. The control benefit is defined as


$$\begin{array}{c} \Delta T(f)=\frac{T_{passive}(f)-T_{controlled}(f)}{T_{passive}(f)}\times 100, \end{array}$$ (1)

where $T_{passive}(f)$ is the displacement transmissibility of the MRE isolator in the passive state and $T_{controlled}(f)$ is the displacement transmissibility under active control. The controller is activated when


$$\begin{array}{c} \Delta T(f)>\Delta T_{\min}, \end{array}$$ (2)

and deactivated when


$$\begin{array}{c} \Delta T(f)\leq \Delta T_{\min}, \end{array}$$ (3)

where $\Delta T_{\min}$ is the minimum control-benefit threshold. In this work, the stopping frequency $f_{s}$ is identified as the first frequency above the resonance region at which the control benefit falls below the selected threshold. This indicates that the passive isolator already provides sufficient attenuation and additional controller action is no longer energy-efficient.

The “Controller Stopping Frequency” is a critical strategic transition parameter that allows the isolator to cease active control interventions, enabling it to naturally dissipate energy and behave as a passive isolator. The identification of this frequency is pivotal in understanding how the controller dynamically responds to varying operating conditions, ensuring optimal performance in structural vibration mitigation. The importance of the Controller Stopping Frequency becomes particularly evident in the development and testing of MRE isolator controllers. By pinpointing the frequencies at which the controller effectively halts the active phase, designers can tailor the controller settings to strategically engage and disengage based on the system’s dynamic response. This adaptability is crucial in real-world scenarios where structural vibrations may vary in frequency and amplitude.

### 5.1. Control strategy implications from observed behavior

The experimental results reveal important control requirements for effective vibration isolation using MRE-based semi-active isolators. As the excitation amplitude increases, the system’s stiffness and natural frequency tend to decrease, which alters the dynamic response. This necessitates a control strategy that can adapt in real time to varying amplitude conditions. Additionally, the isolator exhibits strong nonlinear and hysteretic behavior, particularly near resonance, requiring the controller to be robust against modeling uncertainties. Furthermore, at higher excitation frequencies beyond the Controller Stopping Frequency the system naturally offers sufficient passive isolation. In such cases, continued actuation becomes unnecessary and energy-inefficient. Therefore, an ideal controller should adapt to amplitude changes, compensate for nonlinearities, and intelligently switch off or reduce control effort when active intervention is no longer beneficial. The proposed RBSM controller fulfills these criteria through its hybrid architecture, combining the robustness of sliding mode control with the adaptability of the Radial Basis Function Neural Network.

### 5.2. Control framework

To address the highly nonlinear and amplitude-dependent behavior of the MRE isolator, the proposed control framework adopts a hybrid architecture combining Sliding Mode Control (SMC) and a Radial Basis Function Neural Network (RBFNN). SMC provides robustness to model uncertainties and external disturbances, making it suitable for nonlinear systems. However, its discontinuous control action may induce chattering and struggles to accommodate unmodeled dynamics. To mitigate these issues, an RBFNN is integrated to learn and compensate for unknown nonlinearities in real time. This combination retains the strengths of both methods, achieving high adaptability and reliable control across varying operating conditions.

Within the proposed hybrid framework, the Bouc–Wen model provides the physics-based representation of the MRE hysteresis, whereas the RBF Neural Network functions as an adaptive compensator that estimates the residual nonlinear dynamics arising from hysteretic modeling errors and parameter uncertainties during online operation.

#### 5.2.1. Mathematical modeling of MRE isolator.

The Sliding Mode Controller (SMC), in particular, is a model-based control technique that relies on the system’s differential equations to define the control logic. It requires this mathematical model to accurately compute the equivalent control force and ensure convergence of the system states to the desired trajectory, as established through Lyapunov stability criteria. Without this model, the controller cannot determine the corrective action needed to drive the system to the sliding surface and maintain the desired motion. Hence, a precise model is fundamental to the successful implementation of SMC in handling the nonlinear, amplitude- and frequency-dependent behavior of MRE isolators. To develop an effective and robust control strategy, it is crucial to begin with an accurate mathematical representation of the Magnetorheological Elastomer (MRE) isolator. MRE-based systems exhibit complex nonlinear dynamics arising from viscoelastic damping and hysteresis. These characteristics are influenced by magnetic field strength, excitation amplitude, and frequency, making model fidelity essential for control design.

Fig 7 shows the SDOF model of the MRE isolator incorporating control force *F*(*t*). The system uses Zener and Bouc-Wen elements to represent viscoelastic and hysteretic behavior, respectively. These elements are arranged in parallel, with parameters $\left(k_{1},c,k_{2},z\right)$ varying with magnetic field strength, excitation amplitude, and frequency.

**Fig 7:**
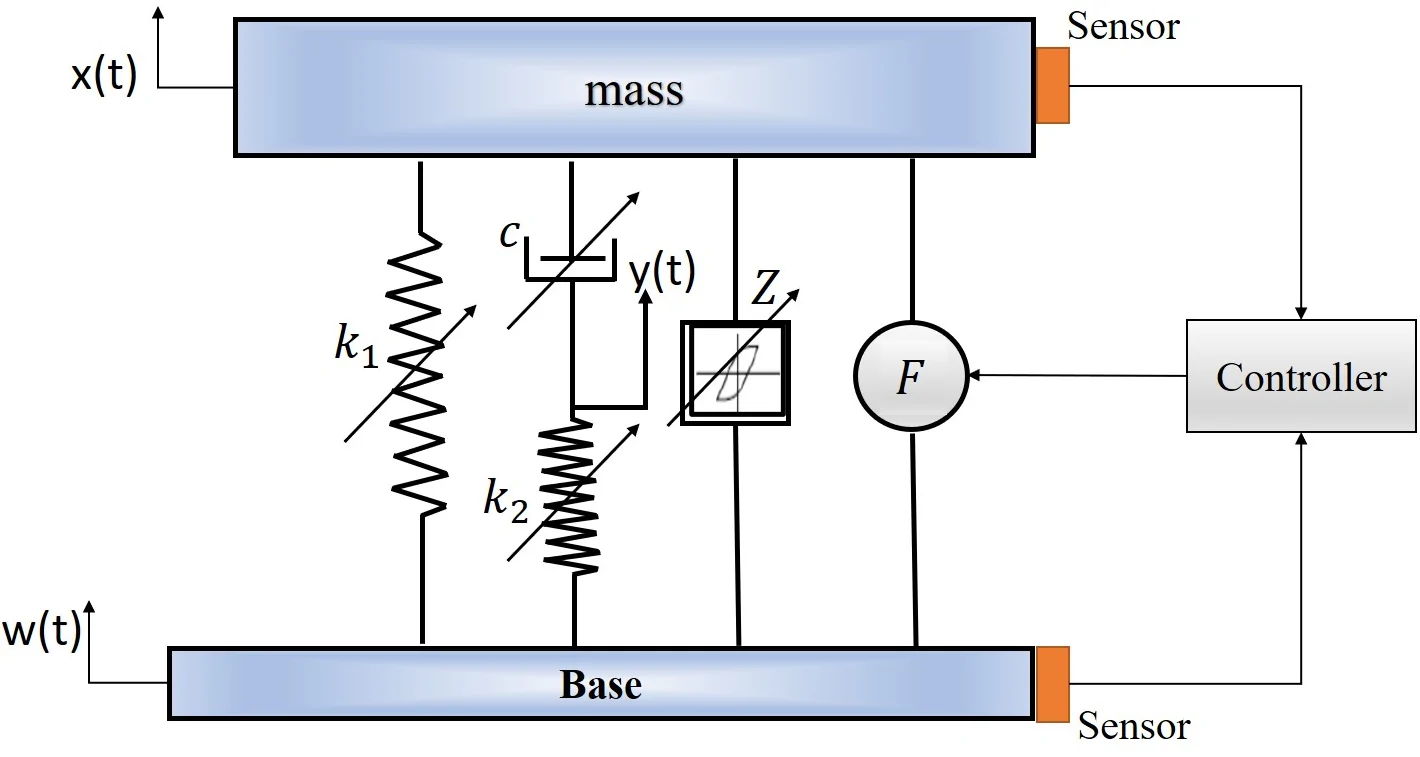
Single-degree-of-freedom (SDOF) representation of the MRE isolator.

The dynamic equations of motion are:


$$\begin{array}{c} m\ddot{x}(t)+k_{1}(x(t)-w(t))+c(\dot{x}(t)-\dot{y}(t))+z(t)+F(t)=0 \end{array}$$ (4)

$$\begin{array}{c} k_{2}(y(t)-w(t))=c(\dot{x}(t)-\dot{y}(t)) \end{array}$$ (5)

The dynamic equations of motion describing the MRE-based vibration isolation system are given in Eqs.(4) and (5). The parameters appearing in these equations are defined as follows.

- *m* denotes the mass of the isolated system or payload supported by the MRE isolator.
- *x*(*t*) represents the displacement of the isolated mass, which corresponds to the response of the system subjected to external excitation.
- *y*(*t*) denotes the internal displacement associated with the viscoelastic element of the isolator.
- *w*(*t*) represents the base excitation displacement, corresponding to the vibration input applied to the base of the isolation system.
- $\dot{x}(t)$ and $\ddot{x}(t)$ denote the velocity and acceleration of the isolated mass, respectively.
- $\dot{y}(t)$ represents the velocity of the internal damping element.
- *k*_1_ denotes the primary stiffness of the MRE isolator, representing the elastic stiffness contribution of the elastomer matrix.
- *k*_2_ represents the stiffness of the auxiliary spring element associated with the internal mechanical structure of the isolator.
- *c* denotes the viscous damping coefficient, which characterizes the energy dissipation due to the viscoelastic behavior of the MRE material.
- *F*(*t*) represents the controllable force generated by the applied magnetic field in the MRE isolator, which modifies the effective stiffness and damping characteristics of the system.
- *z*(*t*) represents the hysteretic restoring force associated with the nonlinear behavior of the MRE material. This force is modeled using the Bouc-Wen hysteresis model.

The nonlinear hysteretic restoring force *z*(*t*) in the MRE isolator is modeled using the Bouc–Wen hysteresis formulation, which has been widely adopted for representing nonlinear hysteretic behavior in structural and smart materials systems [45–47]. The Bouc-Wen model introduces an internal hysteretic variable whose evolution governs the shape and amplitude of the hysteresis loop.

The model parameters adopted in the present study were selected using the experimentally identified dynamic characteristics of the fabricated MRE isolator. In particular, the equivalent stiffness and damping coefficients were determined from the measured frequency-response characteristics, while the observed current-dependent natural-frequency variation was used to validate the parameter selection of the Bouc–Wen model. Therefore, the mathematical model employed for controller design represents the experimentally measured behavior of the MRE isolator.

The evolution equation of the hysteretic variable *z*(*t*) is given by


$$\begin{array}{c} \dot{z}(t)=A\dot{x}(t)-\beta |\dot{x}(t)||z(t){|}^{n-1}z(t)-\gamma \dot{x}(t)|z(t){|}^{n} \end{array}$$ (6)

where *x*(*t*) and $\dot{x}(t)$ denote the displacement and velocity of the isolated mass, respectively.

In this formulation, the parameter *A* controls the linear contribution to the hysteretic evolution, while β and γ govern the nonlinear hysteretic characteristics and influence the shape and smoothness of the hysteresis loop. The exponent *n* is a positive constant that determines the sharpness of the transition between the elastic and hysteretic regions of the response. The hysteretic variable *z*(*t*) represents the nonlinear memory-dependent restoring force of the MRE material and captures *t*he evolution of the hysteresis loop under varying magnetic field strength and excitation conditions. Consequently, *z*(*t*) constitutes one of the dominant nonlinear components governing the dynamic response of *t*he MRE isolator. Although the Bouc–Wen model provides an effective mathematical representation of the hysteretic behavior, discrepancies between the model and the actual MRE response may arise due to parameter variations, material nonlinearities, and operating conditions uncertainties. These uncertainties motivate the incorporation of an adaptive learning mechanism within the proposed control framework.

The Bouc-Wen model is particularly suitable for magnetorheological materials because it can effectively capture the nonlinear and rate-dependent hysteresis observed in experimental responses of MRE-based vibration isolation systems under varying excitation amplitudes and frequencies. Several studies have successfully employed Bouc–Wen based models to describe the dynamic behavior of magnetorheological devices and smart vibration control systems [13,47].

The experimental characterization presented in the previous section provides the dynamic parameters required for the controller development and numerical simulations. Specifically, the experimentally measured natural frequencies, equivalent stiffness, damping ratios, and current-dependent frequency-shift characteristics were used to calibrate the dynamic model of the MRE isolator. These experimentally identified properties were incorporated into the Bouc–Wen-based dynamic model to ensure that the simulated system accurately reproduces the measured vibration behavior under different excitation amplitudes and applied current levels. Consequently, the controller simulations are based on experimentally validated system parameters rather than purely theoretical assumptions, thereby establishing a direct link between the experimental characterization and the subsequent control performance evaluation.

#### 5.2.2 Sliding mode controller design.

In general, there are differences between the response of the mathematical model and the actual response of the MRE isolator. These inconsistencies are caused by a variety of factors, including variations in MRE property due to magnetisation and excitation amplitude. To overcome these inconsistencies and establish a successful controller strategy, a collection of strong controller designs is offered specifically for the MRE isolator. The Sliding Mode Controller methodology is one of the robust controllers for the MRE isolator. The Sliding Mode Controller is distinguished by its ability to perform efficiently for nonlinear systems while remaining robust in the face of parametric uncertainties and external disruptions. Two steps are involved in the design of a Sliding Mode Controller [48]. The first step is to mathematically define the sliding surface so that the MRE isolator can be confined over it in order to get the appropriate system response. The second step is to calculate the expression for switched feedback gains needed to move state trajectory of the plant to the sliding surface. Finally, using the Lyapunov stability theory, the developed controller is proved to be stable.


$$\begin{array}{c} e(t)=x_{d}(t)-x(t),\;\dot{e}(t)={\dot{x}}_{d}(t)-\dot{x}(t) \end{array}$$ (7)

The sliding surface is selected as


$$\begin{array}{c} s=\dot{e}+\alpha e, \end{array}$$ (8)

where α>0 determines the convergence rate of the tracking error. A larger value of α results in faster error convergence but may increase the control effort, whereas a smaller value provides smoother system response at the expense of slower convergence. Therefore, α is selected to achieve a suitable compromise between transient response and control smoothness.

The sliding surface *s*(*t*) is defined as:


$$\begin{array}{c} s(t)=\dot{e}(t)+c_{1}e(t),\;c_{1}>0 \end{array}$$ (9)

This surface captures the system’s tracking error in position and velocity. The control objective is to drive s(t)→0, ensuring that the system state converges to the desired trajectory.

Now, let us express the system dynamics more compactly. Define:


$$\begin{array}{c} f(t)=k_{1}(x(t)-w(t))+c(\dot{x}(t)-\dot{y}(t))+z(t) \end{array}$$ (10)

Then the second-order system becomes:


$$\begin{array}{c} \ddot{x}(t)=-\frac{1}{m}(f(t)+F(t)) \end{array}$$ (11)

#### 5.2.3. Lyapunov stability analysis.

The squared Lyapunov function was selected in this study due to its mathematical simplicity and its effectiveness in analyzing the stability of nonlinear dynamic systems. In sliding mode control and neural network–based adaptive control systems, quadratic Lyapunov candidate functions are commonly used because they ensure positive definiteness and facilitate straightforward derivation of stability conditions.

A typical Lyapunov candidate function is defined as [49,50]


$$\begin{array}{c} V=\frac{1}{2}s^{2} \end{array}$$ (12)

where *s* represents the sliding surface. This quadratic form guarantees that *V* > 0 for all s≠0 and *V* = 0 only when the system state reaches the sliding surface. The derivative of the Lyapunov function can be easily evaluated, which simplifies the stability analysis and enables the derivation of sufficient conditions for asymptotic stability.

Furthermore, the squared Lyapunov function provides a convenient framework for integrating neural network adaptation laws and sliding mode control strategies. This choice allows the stability of the closed-loop system to be rigorously proven while maintaining a computationally efficient control design.

Taking the time derivative of *V*(*t*):


$$\begin{array}{c} \dot{V}(t)=s(t)\dot{s}(t) \end{array}$$ (13)

Differentiating *s*(*t*) using (11):


$$\begin{array}{c} \dot{s}(t)={\ddot{x}}_{d}(t)-\ddot{x}(t)+c_{1}\dot{e}(t)={\ddot{x}}_{d}(t)+c_{1}\dot{e}(t)+\frac{1}{m}(f(t)+F(t)) \end{array}$$ (14)

To ensure $\dot{V}\leq 0$, we impose:


$$\begin{array}{c} \dot{V}(t)=-\eta |s(t)|,\;\eta >0 \end{array}$$ (15)

Since η>0, the Lyapunov function satisfies


$$\begin{array}{c} \dot{V}\leq -\eta |s|<0,\;s\neq 0. \end{array}$$

Therefore, the Lyapunov function is positive definite and its time derivative is negative definite outside the sliding surface. According to Lyapunov’s direct method, all closed-loop signals remain bounded and the tracking error asymptotically converges toward the sliding surface. Furthermore, the adaptive RBFNN continuously estimates the unknown nonlinear dynamics, reducing the magnitude of the uncertainty that must be compensated by the discontinuous switching term. Consequently, the proposed controller preserves the robustness of Sliding Mode Control while improving tracking accuracy and reducing chattering.

The control law consists of an equivalent control component and a discontinuous switching component. The equivalent control compensates for the nominal system dynamics, whereas the switching term provides robustness against modeling uncertainties and external disturbances. The switching gain η is selected to satisfy the reaching condition while avoiding excessive control activity. Consequently, η should be sufficiently large to overcome bounded disturbances but not excessively high, since unnecessarily large switching gains increase control chattering.

This leads to the sliding mode control law:


$$\begin{array}{c} F(t)=m({\ddot{x}}_{d}+\eta \;\text{sgn}(s)+c_{1}\dot{e})-f(t) \end{array}$$ (16)

Here, *F*(*t*) is the control force to be applied to the MRE isolator, and *f*(*t*) encompasses the total system nonlinearities. This formulation ensures that the sliding surface is attractive and the tracking error converges to zero, thereby achieving robust control under uncertainty.

Although the equivalent control law is derived from the mathematical model, its practical implementation relies only on measurable system states and the RBFNN-estimated nonlinear dynamics. Therefore, higher-order state derivatives are not required during online controller execution.

In practical implementation, the control input of the MRE isolator is not the control force directly but the electric current supplied to the electromagnetic coil. The applied current generates a magnetic field, which modifies the mechanical properties of the magnetorheological elastomer and consequently alters the controllable force produced by the isolator.

The magnetic field intensity inside the MRE element is proportional to the input current applied to the coil and can be expressed as


$$\begin{array}{c} H=\frac{NI}{l_{m}} \end{array}$$ (17)

where *H* is the magnetic field intensity, *N* is the number of coil turns, *I* is the applied electric current, and $l_{m}$ is the effective magnetic path length.

The magnetic flux density inside the MRE element can then be written as


$$\begin{array}{c} B=\mu H \end{array}$$ (18)

where μ is the effective magnetic permeability of the MRE composite.

The controllable force generated by the MRE isolator depends on the field-dependent stiffness of the elastomer. Therefore, the control force can be expressed as a function of the applied magnetic field and displacement


$$\begin{array}{c} F_{c}=k(B)x \end{array}$$ (19)

where *k*(*B*) represents the magnetic-field-dependent stiffness of the MRE material.

Since the magnetic flux density *B* is directly related to the applied current *I*, the control force can be represented as


$$\begin{array}{c} F_{c}=k(I)x \end{array}$$ (20)

Thus, the control signal generated by the proposed controller is converted into the required coil current to produce the desired controllable force in the MRE isolator. This relationship allows the semi-active controller to regulate the mechanical response of the isolator through the modulation of the electromagnetic current.

#### 5.2.4. Integration of RBFNN with Sliding Mode Control.

In conventional Sliding Mode Control, high switching gains are generally required to compensate for unknown nonlinearities, resulting in undesirable chattering of the control signal. In the proposed controller, the RBF Neural Network adaptively estimates the unknown nonlinear dynamics online. Consequently, the switching component is required to compensate only for the residual approximation error rather than the complete system uncertainty. This substantially reduces the discontinuous switching action and suppresses control chattering while maintaining the robustness characteristics of Sliding Mode Control.

Although the Sliding Mode Controller (SMC) provides robust performance against system uncertainties and external disturbances, it suffers from a key limitation known as *chattering*, which arises due to the discontinuous nature of the control law. Additionally, the effectiveness of the controller depends on precise knowledge of the nonlinear function *f*(*t*), which encompasses the complex stiffness, damping, and hysteretic forces in the MRE system. However, in real-time applications, such nonlinear dynamics are often unknown or only partially known.

To improve robustness against modeling uncertainties and external disturbances, a Radial Basis Function Neural Network (RBFNN) is integrated with the sliding mode controller. The RBF network is used to approximate unknown nonlinear dynamics, while the sliding mode control law guarantees system robustness and stability. Such hybrid intelligent control strategies combining RBF neural networks and sliding mode control have been widely adopted for nonlinear systems due to their strong approximation capability and robustness [51–53].

An RBFNN is a three-layer feedforward network consisting of:

- An **input layer**, which receives the state vector (typically the tracking error and its derivative),
- A **hidden layer**, which applies nonlinear transformation through Gaussian radial basis functions,
- An **output layer**, which produces an estimate of the unknown nonlinear function.

The approximation model of the nonlinear function *f*(*t*) using RBFNN can be expressed as:


$$\begin{array}{c} f(t)=W^{T}h(x)+\varepsilon \end{array}$$ (21)

where:

- $x=[e(t),\dot{e}(t){]}^{T}$ is the input vector consisting of tracking error and its rate,
- *h*(*x*) is the vector of radial basis functions,
- *W* is the ideal weight vector of the network,
- ε is the bounded approximation error.

The proposed RBF Neural Network adopts a three-layer feedforward architecture consisting of an input layer, a hidden radial basis layer, and a linear output layer. The network input vector is defined as


$$\begin{array}{c} x=[e(t),\dot{e}(t){]}^{T}, \end{array}$$

where *e*(*t*) and $\dot{e}(t)$ denote the tracking error and its first derivative, respectively. These quantities are directly obtained from the measured displacement response and the desired reference trajectory. The hidden layer consists of Gaussian radial basis neurons, whose outputs are expressed as


$$\begin{array}{c} h_{i}(x)=\exp\left(-\frac{\|x-c_{i}{\|}^{2}}{2\sigma_{i}^{2}}\right), \end{array}$$

where $c_{i}$ and $\sigma_{i}$ represent the center and width of the $i^{th}$ radial basis function. The network output is computed as the weighted sum of the hidden-layer responses to approximate the unknown nonlinear dynamics.

The neural network’s estimate of *f*(*t*) is given by:


$$\begin{array}{c} \hat{f}(t)={\hat{W}}^{T}h(x) \end{array}$$ (22)

where $\hat{W}$ is the estimated weight vector.

The input vector of the RBF Neural Network consists only of the tracking error and its first derivative,


$$\begin{array}{c} x=[e(t),\dot{e}(t){]}^{T}, \end{array}$$

which are directly obtained from the measured displacement response of the MRE isolator and the desired reference trajectory. Consequently, the proposed controller does not require higher-order state derivatives or direct measurement of the hysteretic internal variable. Instead, the RBFNN utilizes these measurable signals to estimate the unknown nonlinear dynamics online, thereby providing a practical implementation while avoiding the noise amplification commonly associated with numerical differentiation of higher-order derivatives.

By replacing the unknown *f*(*t*) in the sliding mode control law with its neural network estimate $\hat{f}(t)$, the modified control force becomes:


$$\begin{array}{c} F(t)=m\left({\ddot{x}}_{d}+\eta \;sgn(s)+c_{1}\dot{e}\right)-\hat{f}(t) \end{array}$$ (23)

The estimation error is defined as:


$$\begin{array}{c} \tilde{f}(t)=f(t)-\hat{f}(t)={\tilde{W}}^{T}h(x)+\varepsilon \end{array}$$ (24)

where $\tilde{W}=W-\hat{W}$ is the weight estimation error. It should be emphasized that the proposed RBF Neural Network does not replace the Bouc-Wen hysteresis model employed in the dynamic formulation of the MRE isolator. Instead, the Bouc-Wen model continues to represent the physical hysteretic restoring force through the internal variable *z*(*t*), while the RBFNN adaptively estima*t*es the unknown nonlinear dynamics associated with the modeling uncertainties. Since the hysteretic force represented by *z*(*t*) is embedded within the nonlinear function *f*(*t*), any mismatch between the ma*t*hematical model and the actual hysteretic response is compensated online by the RBFNN. Consequently, the Sliding Mode Controller utilizes the improved estimate $\hat{f}(t)$ to generate a more accurate control action, thereby reducing control chattering and improving robustness against parameter variations, nonlinear hysteresis, and external disturbances.

To ensure stability and convergence, a composite Lyapunov function is constructed as:


$$\begin{array}{c} V=\frac{1}{2}s^{2}+\frac{1}{2\lambda }{\tilde{W}}^{T}\tilde{W} \end{array}$$ (25)

where λ>0 is the neural network adaptation gain.

To ensure that $\dot{V}\leq 0$, the following adaptive update law for the weight vector is used:


$$\begin{array}{c} \dot{\hat{W}}=-\frac{1}{m\lambda }sh(x) \end{array}$$ (26)

This adaptation law enables the neural network to learn the system’s nonlinear behavior online and improve control accuracy while suppressing chattering. The resulting hybrid controller, termed the Radial Basis Sliding Mode (RBSM) controller, combines the robustness of SMC with the adaptability of RBFNN to handle the nonlinear, amplitude-dependent response of the MRE isolator.

The proposed RBFNN operates as an adaptive nonlinear approximator within the RBSM control framework. The initial RBFNN weights are estimated offline using the Levenberg–Marquardt (LM) optimization algorithm based on the available training data. These LM-estimated weights provide the initial network parameters for the RBSM controller. During online controller operation, the network parameters are further adapted according to the Lyapunov-based adaptive law presented in Eq. (26). Therefore, the LM algorithm provides the initial weight estimation, whereas the Lyapunov-based adaptation law enables real-time adjustment of the RBFNN parameters in response to variations in the system dynamics and excitation conditions.

The RBFNN parameters, including the radial basis centers and widths, were selected to provide sufficient approximation capability over the expected operating range of the MRE isolator. The RBFNN employs a 2–10–1 architecture consisting of two input neurons corresponding to the tracking error \(e(t)\) and its derivative \(\dot e(t)\), ten Gaussian radial basis neurons in the hidden layer, and one output neuron representing the estimated nonlinear dynamics \(\hat f(t)\). The number of hidden neurons was selected through preliminary tuning to achieve a suitable compromise between approximation accuracy and computational complexity. During controller operation, the adaptive weight update law continuously minimizes the approximation error of the unknown nonlinear dynamics, enabling real-time compensation of modeling uncertainties.

### 5.3. Performance assessment of the developed controller

It should be noted that the simulation results presented in this section are not independent of the experimental characterization. The controller performance is evaluated using the experimentally calibrated MRE dynamic model, allowing the numerical simulations to reflect the actual stiffness variation, damping characteristics, and frequency-shift behavior observed during experimental testing.

The objective of the present simulation study is not to benchmark the proposed controller against different control algorithms, but rather to evaluate its capability to adapt to varying excitation amplitudes and frequencies using the experimentally calibrated MRE model. The presented results therefore demonstrate the adaptive behavior of the proposed Hybrid RBFNN–Sliding Mode Controller in maintaining effective vibration isolation across the investigated operating conditions, including the transition between active and passive control at the controller stopping frequency.

The RBSM control architecture used in the simulations is illustrated in Fig 8, showing the integration of the Sliding Mode Controller and the adaptive neural network. The assessment of a radial basis Sliding Mode Controller for Magnetorheological Elastomer (MRE) isolators in simulation is pivotal for understanding its performance across varying amplitudes of excitation and frequencies. The MRE isolator response exhibits a nuanced dependence on both amplitude and frequency. The controller’s examination under diverse conditions serves to elucidate its adaptability and efficacy in regulating the dynamic behavior of the MRE isolator. Particularly noteworthy is the controller’s role in semi-active control, where it facilitates the transition from an active to a passive phase at specific frequencies, commonly referred to as the transition or Controller Stopping Frequency. This transition frequency holds significant importance as it allows the controller to actively engage and optimize the isolator’s response within a specific frequency range, seamlessly transitioning to a passive state beyond this critical frequency. Through systematic testing, the controller’s ability to navigate and exploit the frequency-dependent nature of MRE isolators becomes evident, signifying its potential to enhance structural vibration mitigation across a broad spectrum of amplitudes and frequencies. This comprehensive evaluation sheds light on the intricate dynamics of the controller, providing valuable insights for optimizing its application in the realm of MRE isolator control.

**Fig 8:**
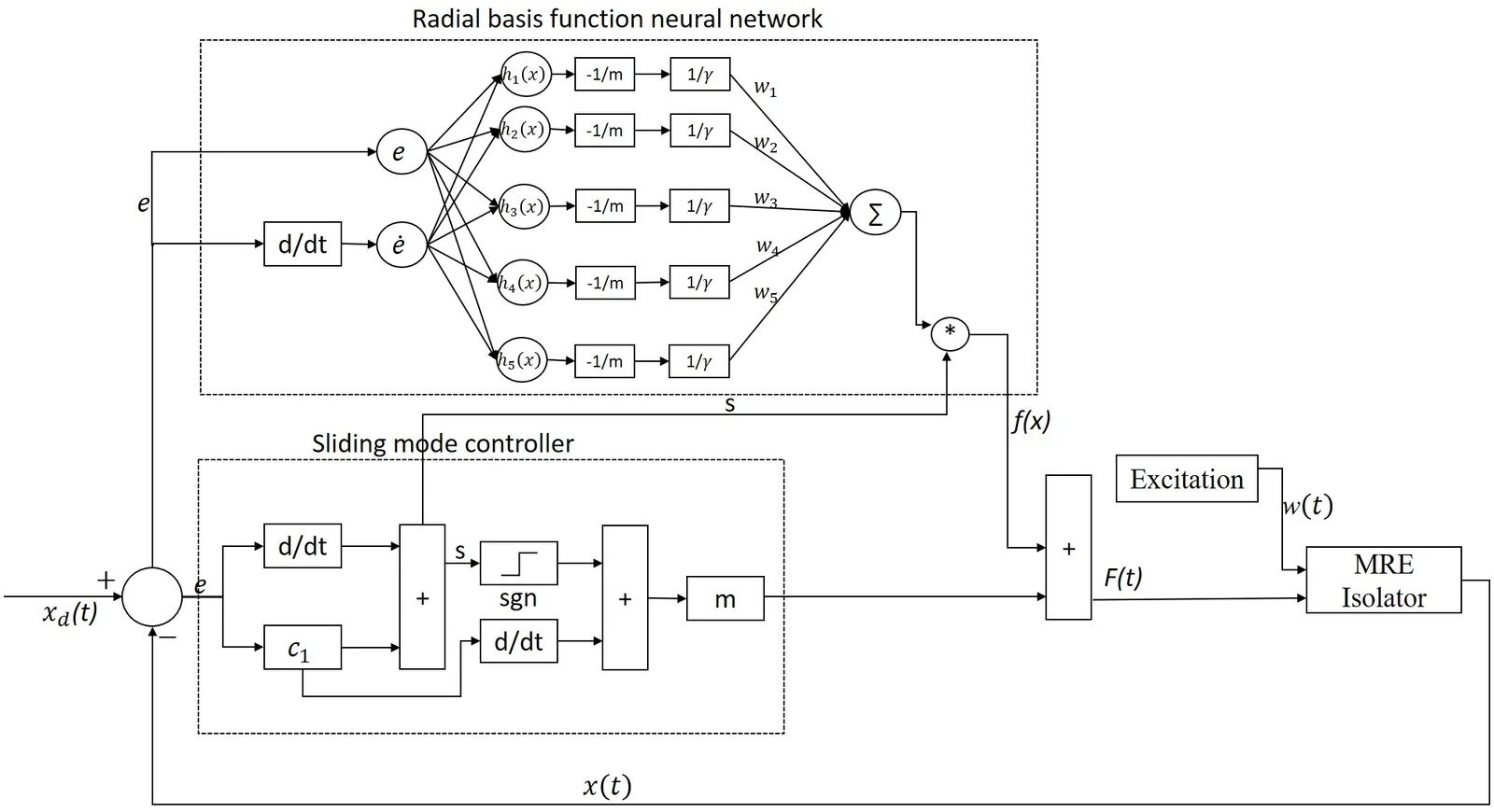
Block diagram of the RBSM control scheme.

The simulation conditions were strategically selected to evaluate the controller’s performance under both maximum vibration and minimal control zones. The maximum vibration zone, occurring near the system’s natural frequency, represents conditions where active control is critical to suppress resonance-induced amplification. In contrast, the minimal control zone corresponds to frequencies beyond the controller stopping point, where the system exhibits adequate passive isolation and active intervention is unnecessary. Evaluating controller performance across these zones ensures both robust vibration mitigation and energy-efficient operation. To represent the maximum isolation demand, five test cases were chosen at the system’s natural frequency (28 Hz), where the transmissibility is highest and active control is most critical. These cases varied in excitation amplitudes between 0.00125 m and 0.00225 m to test amplitude-dependent controller response. The remaining five conditions were selected based on the identified controller stopping frequencies (39.5 Hz, 35.16 Hz, 34.52 Hz, 34.25 Hz, and 33.78 Hz), which were derived from transmissibility tests showing a natural transition from active to passive behavior. At these frequencies, the isolator already exhibits sufficient passive damping, and the controller is expected to disengage. These stopping frequencies decrease with increasing amplitude, highlighting the nonlinear behavior of the MRE and necessitating adaptive control. This selection allows a comprehensive evaluation of the RBSM controller’s ability to engage effectively at resonance, and to withdraw intelligently when control is no longer beneficial–-thereby reducing unnecessary actuation and energy consumption.

The choice of different amplitudes and frequencies is crucial for a comprehensive evaluation of the controller’s performance. Varying amplitudes represent different excitation magnitudes that the MRE isolator may encounter in real-world scenarios, and analyzing the controller’s response to these variations provides insights into its adaptability. Additionally, the consideration of different frequencies, including the stopping frequency, is essential. The control stopping frequency represents the transition point at which the controller shifts from an active to a passive state. Understanding and optimizing this transition point are vital for achieving an effective balance between vibration isolation and energy efficiency. This comprehensive analysis of diverse amplitude and frequency scenarios ensures a robust evaluation of the RBSM controller’s efficacy in real-world applications.

The performance characteristics of the MRE isolator at different excitation frequencies and amplitudes are summarized in Table 3. These values are obtained from the experimental study conducted in our previous work [44], where the dynamic response characteristics of the MRE isolator were investigated under varying operating conditions. The weight of the neural network is chosen in such a way that the Sliding Mode Controller produces the required control output for the desired vibration isolation. The neural network automatically changes its weights as the amplitude of excitation changes, causing the controller output from the Sliding Mode Controller to change and achieve the required isolation.

**Table 3:** Performance characteristics of the MRE isolator at different frequencies and amplitudes. Data taken from our previous work [44].

Frequency (Hz)	Input displacement (m)	Transmissibility without current (0 A)	Transmissibility at 3 A	Isolation (%)
28.00	0.00125	2.47	1.09	55.8
39.50	0.00125	1.19	1.19	0.0
28.00	0.00150	2.47	1.39	51.8
35.16	0.00150	1.68	1.68	0.0
28.00	0.00175	2.47	1.45	41.2
34.52	0.00175	1.77	1.77	0.0
28.00	0.00200	2.47	1.47	40.4
34.25	0.00200	1.81	1.81	0.0
28.00	0.00225	2.47	1.51	38.8
33.78	0.00225	1.88	1.88	0.0

The structure of a RBFNN is 2-10-1. (two-neuron input layer, 10-neuron hidden layer, and one-neuron output layer). Ten scenarios are studied, each with a different frequency and magnitude of excitation (Ten cases are listed in Table 3). Because the input vector size was 10, the hidden layer takes into account 10 neurons. The weight of the linear layer and bias are selected so that only one neuron is engaged for each error and error rate input. The weights of the neural network and biases are chosen as


$$\begin{array}{c} W^{1}=\left[\begin{array}{c} \begin{array}{cc} 0.00125 & 0.21991 \end{array}\\ \begin{array}{cc} 0.00150 & 0.26389 \end{array}\\ \begin{array}{c} \begin{array}{cc} 0.00175 & 0.30787 \end{array}\\ \begin{array}{cc} 0.00125 & 0.31023 \end{array}\\ \begin{array}{c} \begin{array}{cc} 0.00150 & 0.33137 \end{array}\\ \begin{array}{cc} 0.00225 & 0.47755 \end{array}\\ \begin{array}{c} \begin{array}{cc} 0.00200 & 0.35185 \end{array}\\ \begin{array}{cc} 0.00175 & 0.37956 \end{array}\\ \begin{array}{c} \begin{array}{cc} 0.00225 & 0.39584 \end{array}\\ \begin{array}{cc} 0.00200 & 0.43039 \end{array} \end{array} \end{array} \end{array} \end{array} \end{array}\right]b^{1}=\left[\begin{array}{c} 0.83255\\ 0.83255\\ 0.83255\\ 0.83255\\ \begin{array}{c} 0.83255\\ 0.83255\\ \begin{array}{c} 0.83255\\ 0.83255\\ \begin{array}{c} 0.83255\\ 0.83255 \end{array} \end{array} \end{array} \end{array}\right]W^{2}=10^{12}{\left[\begin{array}{c} -1.469\\ \begin{array}{c} 4.154\\ -4.823\\ \begin{array}{c} 1.187\\ 0\\ \begin{array}{c} -1.552\\ 3.680\\ \begin{array}{c} -5.163\\ \begin{array}{c} -9.782\\ 4.966 \end{array} \end{array} \end{array} \end{array} \end{array} \end{array}\right]}^{T}b^{2}=\left[260944770.7\right] \end{array}$$ (27)

The weights of the network are typically estimated through a training process. The process involves adjusting the weights iteratively to minimize the difference between the network’s predicted output and the actual target output. The Levenberg–Marquardt algorithm plays a crucial role in this weight adjustment process.

The weights and biases of the neural network were estimated through a supervised training process using the Levenberg–Marquardt (LM) optimization algorithm. In the proposed controller, the neural network uses the displacement error and the error rate as input variables, while the network output represents the desired additional control force required for vibration suppression.

The displacement error and error rate are defined as


$$\begin{array}{c} e(t)=x_{d}(t)-x(t) \end{array}$$ (28)

$$\begin{array}{c} \dot{e}(t)={\dot{x}}_{d}(t)-\dot{x}(t) \end{array}$$ (29)

where $x_{d}(t)$ represents the desired displacement and *x*(*t*) represents the measured displacement of *t*he system.

The neural network is trained to estimate the desired additional control force $F_{d}(t)$ required to regulate the vibration of the system. During the training process, the difference between the desired control force and the neural network predicted force is minimized using the mean squared error (MSE) cost function:


$$\begin{array}{c} E=\frac{1}{2}\sum_{i=1}^{N}(F_{d,i}-{\hat{F}}_{i}{)}^{2} \end{array}$$ (30)

where $F_{d,i}$ denotes the desired extra control force and ${\hat{F}}_{i}$ represents the neural network predicted force.

The LM algorithm iteratively updates the neural network weight vector 𝐰 according to


$$\begin{array}{c} {𝐰}_{k+1}={𝐰}_{k}-(J^{T}J+\mu I{)}^{-1}J^{T}𝐞 \end{array}$$ (31)

where *J* is the Jacobian matrix of partial derivatives of the network error with respect to the weights, μ is the damping parameter controlling convergence, and 𝐞 is the vector of prediction errors. Through this iterative process, the neural network parameters are adjusted until the prediction error between the desired and estimated control force is minimized.

With the neural network weights chosen, the RBSM controller was implemented on the MRE isolator, with sliding mode gains *c*_1_ and η set to 130 and 0.1, respectively. The selected value of η = 0.1 resulted in negligible/reduced chattering in the simulated control response.

To evaluate the performance of the proposed controller under varying excitation conditions, both the amplitude and frequency of the sinusoidal input applied to the MRE isolator were systematically varied. The effectiveness of the controller was assessed by comparing the displacement response of the system with and without control action.

Fig 9(a) shows the displacement response of the MRE isolator, while Fig 9(b) illustrates the corresponding control force generated by the controller. To investigate the influence of excitation conditions on controller performance, the input amplitude and frequency were varied during the simulation.

**Fig 9:**
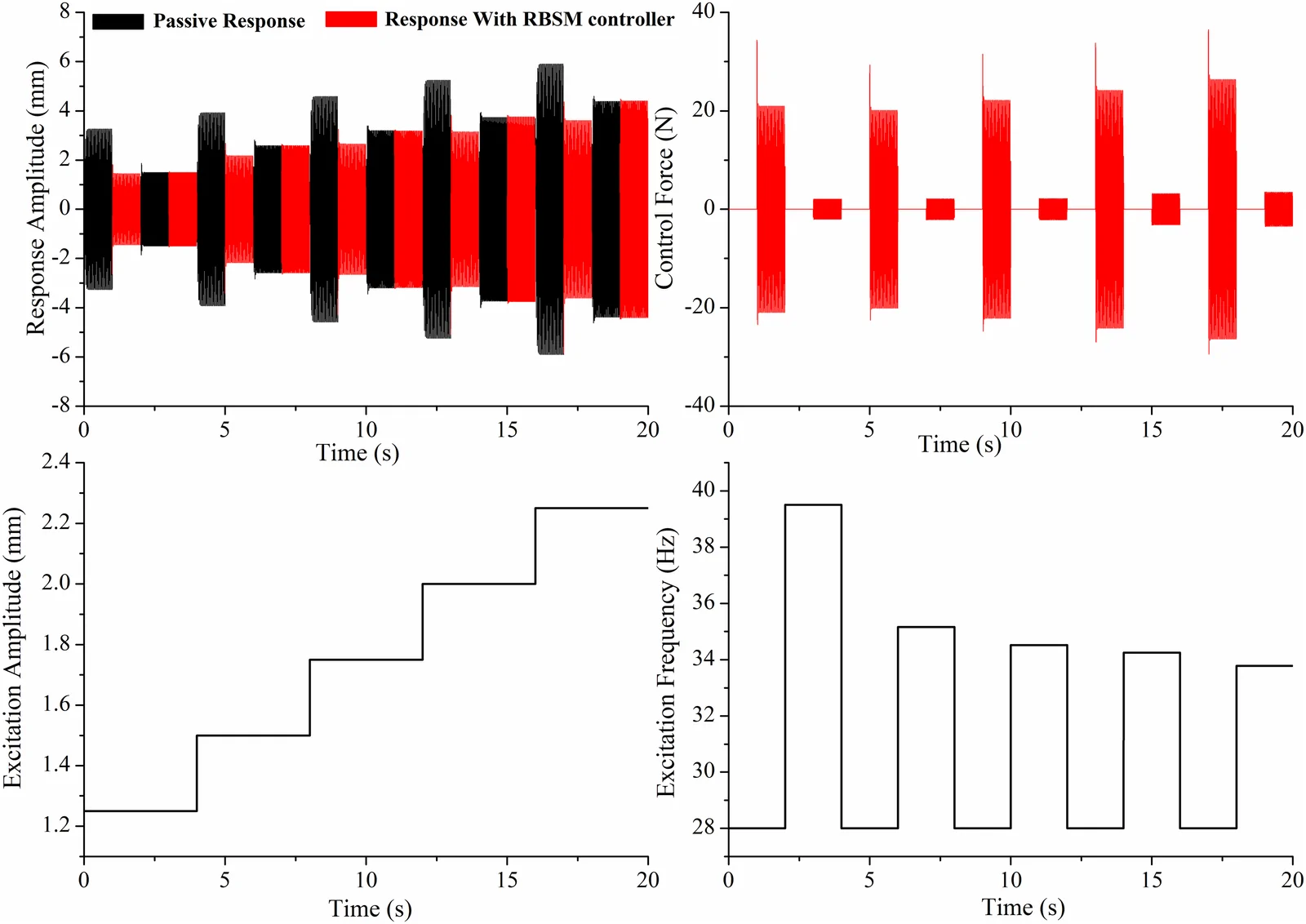
Performance of the tuned RBSM controller: (a) Response of MRE isolator with and without controller (b) Control Force of the controller (c) Variation of excitation amplitude with respect to time (d) Variation of excitation frequency with respect to time.

The excitation amplitude was changed from 0.00125 m to 0.00225 m in discrete steps every 4 s over a total simulation duration of 20 s, as shown in Fig 9(c). Simultaneously, the excitation frequency was varied every 2 s over the same time interval, as illustrated in Fig 9(d). For each test condition, the controller was turned off for 1 s and then reactivated for the following 1 s to enable comparison between controlled and uncontrolled system responses.

The maximum vibration isolation of approximately 55.8% was observed between 2 s and 4 s, corresponding to an excitation amplitude of 0.00125 m and a frequency of 28 Hz. The highest control effort was also recorded during this period (3 s to 4 s), as shown in Fig 9(b). As the excitation amplitude increased from 0.00125 m to 0.00225 m, the vibration isolation at the natural frequency (28 Hz) gradually decreased to 38.8%, demonstrating the controller’s adaptability to amplitude variations.

At excitation frequencies of 39.5 Hz, 35.16 Hz, 34.52 Hz, 34.25 Hz, and 33.78 Hz, the system achieved sufficient passive vibration isolation. Consequently, the controller automatically disengaged, resulting in negligible additional vibration suppression (approximately 0% improvement). These frequencies therefore represent the *controller stopping region*, where active control is not required.

This behavior demonstrates the intelligent switching capability of the proposed controller between active and passive modes depending on the operating conditions. Overall, the results confirm that the controller is responsive to variations in both excitation amplitude and frequency and can effectively distinguish between operating regions that require active control and those where passive isolation is sufficient.

## 6. Conclusions

This study presented a hybrid adaptive control strategy for vibration suppression in a magnetorheological elastomer (MRE)-based vibration isolation system. The proposed controller integrates sliding mode control with a radial basis function (RBF) neural network to address the nonlinear and frequency-dependent characteristics of the MRE isolator. The neural network component estimates unknown nonlinear dynamics in real time, while the sliding mode control framework ensures stable system behavior and effective disturbance rejection. From a scientific perspective, the proposed framework demonstrates how a physics-based hysteresis model and a data-driven adaptive learning algorithm can be effectively integrated within a robust nonlinear control architecture. Rather than replacing the underlying Bouc–Wen hysteresis model, the RBFNN compensates for the residual nonlinear dynamics and modeling uncertainties, thereby improving controller adaptability while preserving the physical interpretability of the system model. This hybrid strategy provides a practical methodology for controlling nonlinear smart-material-based vibration isolation systems operating under varying excitation conditions. The effectiveness of the proposed RBSM controller was evaluated through simulation studies under varying excitation amplitudes and frequencies. The results demonstrate that the proposed controller significantly improves vibration isolation performance across the investigated frequency range of 15–80 Hz. In particular, the controller effectively suppresses vibration near the resonance region where vibration amplification is typically most severe.

The proposed RBFNN–SMC controller achieved a maximum vibration reduction of 55.8% at the resonance frequency under the investigated operating conditions. In addition, the adaptive capability of the RBF neural network enables the controller to automatically adjust its control action in response to changes in excitation frequency and amplitude, resulting in improved vibration attenuation over the investigated operating range.

Furthermore, the controller maintains stable performance under varying operating conditions, demonstrating its capability to handle the nonlinear behavior of the MRE-based isolation system. The results highlight the potential of integrating intelligent learning techniques with robust control methods for advanced adaptive vibration control applications.

Overall, the proposed hybrid RBSM control framework provides an effective and practical solution for adaptive vibration suppression in MRE-based vibration isolation systems. Future work will focus on the experimental implementation of the proposed controller and its evaluation under more complex excitation environments, including multi-frequency, broadband, and random vibration inputs, to further assess its robustness and applicability to real-world engineering systems.

## Nomenclature

### Roman Symbols

**Table:** 

Symbol	Description	Unit
*m*	Mass of the isolated system (payload)	kg
*x*	Displacement of isolated mass	m
$\dot{x}$	Velocity of isolated mass	m s^–1^
$\ddot{x}$	Acceleration of isolated mass	m s^–2^
$x_{d}$	Desired displacement	m
${\dot{x}}_{d}$	Desired velocity	m s^–1^
${\ddot{x}}_{d}$	Desired acceleration	m s^–2^
*y*	Internal displacement of viscoelastic element	m
$\dot{y}$	Velocity of internal viscoelastic element	m s^–1^
*w*	Base excitation displacement	m
*k*_1_	Primary stiffness of the MRE isolator	N m^–1^
*k*_2_	Auxiliary spring stiffness	N m^–1^
*c*	Viscous damping coefficient	N s m^–1^
*F*(*t*)	Control force	N
$F_{c}$	Controllable force generated by the MRE	N
$F_{d}$	Desired control force	N
$\hat{F}$	Neural network predicted control force	N
*H*	Magnetic field intensity	A m^–1^
*B*	Magnetic flux density	T
*I*	Coil current	A
*N*	Number of coil turns	–
$l_{m}$	Effective magnetic path length	m
*A*	Bouc–Wen model parameter	–
*n*	Bouc–Wen exponent	–
*z*	Hysteretic restoring force	N
*e*	Tracking error	m
*s*	Sliding surface	–
*V*	Lyapunov function	–
*E*	Mean squared error	–
*J*	Jacobian matrix	–
*f*(*t*)	Unknown nonlinear system dynamics	–
$\hat{f}(t)$	Estimated nonlinear dynamics	–
$\tilde{f}(t)$	Nonlinear dynamics estimation error	–
*W*	Ideal neural network weight vector	–
$\hat{W}$	Estimated neural network weight vector	–
$\tilde{W}$	Neural network weight estimation error	–
*h*(*x*)	Radial basis function vector	–

### Greek Symbols

**Table:** 

Symbol	Description	Unit
μ	Magnetic permeability	H m^–1^
$\mu_{r}$	Relative magnetic permeability	–
β	Bouc–Wen hysteresis parameter	–
γ	Bouc–Wen hysteresis parameter	–
λ	Neural network adaptation gain	–
η	Sliding mode gain	–
ε	Neural network approximation error	–

### Abbreviations

**Table:** 

Abbreviation	Description
AI	Artificial Intelligence
ANN	Artificial Neural Network
AWG	American Wire Gauge
CIP	Carbonyl Iron Particles
FRF	Frequency Response Function
LM	Levenberg–Marquardt
MRE	Magnetorheological Elastomer
MSE	Mean Squared Error
PID	Proportional–Integral–Derivative
RBF	Radial Basis Function
RBFNN	Radial Basis Function Neural Network
RBSM	Radial Basis Sliding Mode Controller
SDOF	Single Degree of Freedom
SMC	Sliding Mode Control
