# Supplementary material for: Hybrid RBF neural network–based sliding mode control for a magnetorheological elastomer isolator under variable frequency and amplitude excitations
Source: PLoS One. 2026 Oct 9;21(10):e0360069. doi: 10.1371/journal.pone.0360069 (PMC13652330; doi:10.1371/journal.pone.0360069)
Supplement: Supplementary file 1 [file pone.0360069.s001.doc]

The manuscript presents an interesting hybrid control strategy that combines a Radial Basis Function Neural Network (RBFNN) with a Sliding Mode Controller (SMC) for the semi-active vibration isolation of a magnetorheological elastomer (MRE) isolator. Given the inherent nonlinearities and hysteresis of MRE materials, exploring intelligent control combinations is highly meaningful for engineering applications. The manuscript is well-organized, and the preliminary results are encouraging. However, the manuscript could be significantly strengthened by refining the title, clarifying the core novelty, providing more details on controller feasibility, and expanding the baseline comparisons. A Major Revision is recommended to help the authors elevate the quality and impact of their work.

1 The current title is somewhat long and contains introductory phrasing (e.g., "A Study on the Implementation of..."), which can be streamlined to increase its academic readability and directness.

2 The current manuscript presents a solid engineering application, but the unique scientific contribution and specific advantages of this hybrid framework could be highlighted more prominently.

3 In standard sliding mode derivations, equivalent control terms often require high-order state derivatives. It would be helpful to clarify the practical execution of the RBFNN inputs.

4 The core challenge of MRE control lies in its nonlinear shear hysteresis loop, typically governed by the evolutionary internal variable *z* in the Bouc-Wen model. The text would benefit from a clearer explanation of how the RBFNN interacts with this specific hysteretic behavior.

5 To better demonstrate the advantages of the proposed "Hybrid RBF-SMC", the current comparison mainly focuses on the Passive state and standard SMC.

6 The title highlights "Variable Frequency and Amplitude Excitations". While the single-frequency variations are well-presented, evaluating the system under more complex conditions would add significant value.

7 To enhance readability, a dedicated Nomenclature list defining all Roman symbols, Greek symbols, and abbreviations would be a wonderful addition before the Introduction or in an Appendix.

8 Since intelligent control of smart materials is a rapidly evolving field, the authors are encouraged to include a few recent references from the past three years (2023-2026) regarding advanced control schemes for smart dampers/isolators to better reflect the latest state-of-the-art context.
