# Supplementary material for: Hybrid RBF neural network–based sliding mode control for a magnetorheological elastomer isolator under variable frequency and amplitude excitations
Source: PLoS One. 2026 Oct 9;21(10):e0360069. doi: 10.1371/journal.pone.0360069 (PMC13652330; doi:10.1371/journal.pone.0360069)
Supplement: Supplementary file 2 [file pone.0360069.s003.doc]

**Reviewer 1**

1.The manuscript reports a maximum vibration reduction of approximately 65%–70% in the abstract, about 55.8% in the results section, and 55%–60% in the conclusion. Since these values represent the main performance claim of the study, the authors should unify the evaluation criteria and calculation basis, and ensure consistency across the abstract, results, and conclusion.

**Response:**

The manuscript has been carefully revised to ensure consistency in the reported vibration reduction values. The vibration reduction is now consistently evaluated using the percentage reduction in displacement transmissibility defined in Eq. (1). Accordingly, the Abstract, Results, and Conclusion have all been revised to report the same maximum vibration reduction of **55.8%**, corresponding to the experimental conditions summarized in Table 3. These revisions ensure a uniform evaluation criterion and consistent reporting throughout the manuscript.

2. The link between experimental results and control simulations is not sufficiently clear. The manuscript experimentally investigates the current-dependent and amplitude-dependent characteristics of the MRE isolator. However, the subsequent controller validation is mainly based on simulations. The authors should clarify how the experimentally obtained natural frequencies, stiffness, damping ratios, and frequency-shift characteristics are incorporated into the dynamic model and controller design.

**Response:**

In the present study, the primary objective was to investigate the effectiveness of the proposed Radial Basis Sliding Mode (RBSM) control strategy in mitigating vibrations under deterministic harmonic excitation with varying frequencies. The excitation frequency range of 15–80 Hz was selected based on the operational conditions of the experimental vibration test setup used for evaluating the displacement transmissibility characteristics of the MRE isolator. The proposed controller is designed to adaptively regulate the control input according to the variation in excitation frequency, which allows the isolator to transition effectively between the active and passive operating states. The results presented in the manuscript demonstrate the capability of the proposed control strategy to reduce vibration transmissibility across the considered frequency band. The performance of the semi active MTRE isolator is assessed based on the simulation results.

However, a detailed investigation of the controller performance under needs to be carried out under random excitation inputs would require additional modelling of stochastic disturbances and extended simulation studies. Such an analysis, while valuable, is beyond the scope of the current manuscript. This would be addressed in the separate communication.

As highlighted by the reviewer, in the revised manuscript, the relationship between the experimental characterization and the subsequent controller simulations should be explained more clearly. Additionally, manuscript has been revised to clarify that the experimentally obtained dynamic characteristics of the MRE isolator, including the natural frequency, equivalent stiffness, damping ratio, and current-dependent frequency-shift behavior, were used to parameterize and validate the dynamic model employed in the controller simulations. The Bouc–Wen model parameters and the equivalent dynamic properties were selected to reproduce the experimentally observed behavior of the MRE isolator under different excitation amplitudes and coil current levels. Consequently, the simulation results are based on a dynamic model calibrated using the experimentally measured characteristics rather than purely theoretical assumptions.

3. Insufficient description of the RBF neural network.
The RBF neural network is used to approximate unknown nonlinearities, but important details such as the network architecture, input variables, number of hidden nodes, source of training data, and training error are not sufficiently provided. The authors should supplement these parameters and the training procedure to improve the reproducibility of the proposed method.

**Response:**

The additional implementation details of the RBF Neural Network improve the reproducibility of the proposed controller. Accordingly, the manuscript has been revised to provide a more detailed description of the RBFNN architecture, including the network inputs, hidden layer configuration, radial basis activation function, network output, and adaptive weight update mechanism. Furthermore, the manuscript now clarifies that the RBFNN operates as an online adaptive approximator within the control loop, where the network weights are updated according to the Lyapunov-based adaptation law using measured system states, rather than through offline supervised training. These additions clarify the implementation procedure and improve the reproducibility of the proposed method.

4. Further clarification of the sliding mode controller design is needed.
Although the manuscript provides the parameters of the sliding mode controller, the rationale for parameter selection, the stability proof, and the measures used to suppress chattering are not adequately explained. The authors should provide a clearer derivation of the control law, a more complete Lyapunov stability analysis, and the specific approach adopted to mitigate chattering.

**Response:**

The design methodology of the proposed Sliding Mode Controller explained in greater detail. Accordingly, the manuscript has been revised to clarify the rationale for selecting the sliding surface and controller parameters, the derivation of the control law, and the Lyapunov-based stability analysis. In addition, the manuscript now explains how the adaptive RBF Neural Network estimates the unknown nonlinear dynamics, thereby reducing the discontinuous switching action required by the Sliding Mode Controller and mitigating control chattering. These additions improve the transparency and reproducibility of the proposed control strategy.

5. The definition of the controller stopping frequency is not rigorous enough.
The manuscript proposes that the controller automatically switches off at certain frequencies, but the criterion for determining the stopping frequency is not clearly defined. The authors should provide a clear mathematical or engineering criterion, for example based on displacement transmissibility, control benefit, or frequency-response characteristics, to determine when the controller should be activated or deactivated.

**Response:**

The controller stopping frequency is a unique term coined by the author to distinguish between the active and the passive switch over state of the controller. Stopping frequency is a limiting frequency which could eliminate the risk of instability of the system due to continued active state. Without knowing the stopping frequency, the controller will not have the clue about the when the isolator should be passive to prevent the system reaching the resonance of the active state. Since the cut off frequency is unique for a specific isolator, developing a generalized mathematical approach would not be feasible. However as stated by the reviewer, the controller stopping frequency is defined using a clearer engineering criterion. Accordingly, the manuscript has been revised to define the stopping frequency based on the displacement transmissibility response of the MRE isolator. In the revised manuscript, the controller is activated when the controlled state provides a measurable reduction in displacement transmissibility compared with the passive state. Conversely, the controller is deactivated when the improvement in transmissibility becomes negligible or when passive isolation is already sufficient. This criterion provides a clear basis for determining the controller activation and deactivation regions.

6. The comparative validation is still insufficient.
The manuscript claims that the proposed controller outperforms the conventional sliding mode controller, but the comparison and evaluation metrics remain limited. The authors should provide a more systematic comparison with passive isolation, constant-current control, and conventional sliding mode control, using quantitative indicators such as peak response, RMS response, or displacement transmissibility.

**Response:**

We sincerely thank the reviewer for this valuable suggestion. We respectfully clarify that the present manuscript does not claim that the proposed Hybrid RBFNN–SMC controller outperforms a conventional Sliding Mode Controller. The primary objective of this work is to develop and demonstrate a hybrid adaptive control framework capable of maintaining effective vibration isolation under simultaneously varying excitation frequency and excitation amplitude, rather than to perform a comprehensive benchmarking study against multiple control algorithms.

The experimental characterization presented in this study establishes the current-dependent and amplitude-dependent dynamic properties of the fabricated MRE isolator. Based on these experimentally identified characteristics, a Hybrid RBF Neural Network–Sliding Mode Controller is developed to adaptively compensate for the nonlinear and hysteretic behavior of the isolator over the investigated operating range. Consequently, the simulation and experimental results are intended to demonstrate the controller's ability to maintain vibration reduction and stable operation under varying operating conditions, rather than to establish superiority over other control strategies.

For this reason, a detailed comparison with conventional Sliding Mode Control, constant-current control, fuzzy control, or other intelligent controllers has not been included in the present work. Such comparisons would require the implementation, parameter tuning, and validation of multiple controller architectures under identical operating conditions, which would substantially broaden the scope of the study.

The manuscript has been revised to clarify that the principal contribution of this work is the development and validation of an adaptive Hybrid RBFNN–SMC framework for frequency- and amplitude-varying excitation, and a comprehensive comparative study with conventional Sliding Mode Control and other advanced intelligent control methods has been identified as an important direction for future research.

**Reviewer 2**

1. (R) A general review by the authors is necessary, focusing on writing, correcting errors such as “charac-teristics”, ”uncertain-ties”, “model-ing” and others.

**Response:**

The entire manuscript has been carefully proofread and revised to improve the quality of the writing. Typographical errors, word breaks, grammatical inconsistencies, and formatting issues—including examples such as "charac-teristics", "uncertain-ties", and "model-ing"—have been corrected throughout the manuscript. In addition, the text has been edited to improve readability, consistency, and overall language quality.

2. (R) There should be a more in-depth discussion of the practical and realistic application of the magnetorheological elastomer isolators.

**As suggested by the reviewer, the introduction section is modified by adding additional information on the practical and realistic applications of MRE**

**Reviewer 3**

1 The current title is somewhat long and contains introductory phrasing (e.g., "A Study on the Implementation of..."), which can be streamlined to increase its academic readability and directness.

**Response:**

We sincerely thank the reviewer for this valuable suggestion. We agree that the original title was unnecessarily lengthy and contained introductory phrasing that reduced its directness. Accordingly, the title has been revised to improve its academic readability while preserving the technical scope of the study.

**Revised Title:**

*Hybrid RBF Neural Network-Based Sliding Mode Control for a Magnetorheological Elastomer Isolator under Variable Frequency and Amplitude Excitations*

2 The current manuscript presents a solid engineering application, but the unique scientific contribution and specific advantages of this hybrid framework could be highlighted more prominently.

**Response:**

Accordingly, the Introduction has been revised to explicitly highlight the novelty of the proposed approach. Unlike conventional Sliding Mode Control, the proposed framework integrates an adaptive RBF Neural Network with a physics-based Bouc–Wen hysteresis model to compensate for nonlinear modeling uncertainties while preserving the robustness of Sliding Mode Control. Furthermore, the controller is specifically designed for MRE isolators operating under simultaneously varying excitation frequency and amplitude, allowing adaptive compensation of hysteretic nonlinearities without requiring an exact system model. These scientific contributions and their advantages over conventional control strategies have been clarified in both the Introduction and the Conclusion.

3 In standard sliding mode derivations, equivalent control terms often require high-order state derivatives. It would be helpful to clarify the practical execution of the RBFNN inputs.

**Response:**

The practical implementation of the RBF Neural Network inputs is explained more clearly. In the proposed controller, the RBFNN uses only the tracking error and its first derivative as input variables. These quantities are directly obtained from the measured displacement response and the desired reference trajectory, and therefore no higher-order state derivatives are required for online implementation. The RBFNN adaptively estimates the unknown nonlinear dynamics based on these measurable signals, while the Sliding Mode Controller utilizes this estimate to generate the control action. Additional clarification has been added to the manuscript to explain the practical realization of the RBFNN inputs.

4 The core challenge of MRE control lies in its nonlinear shear hysteresis loop, typically governed by the evolutionary internal variable *z* in the Bouc-Wen model. The text would benefit from a clearer explanation of how the RBFNN interacts with this specific hysteretic behavior.

**Response:**

The interaction between the proposed RBF Neural Network and the nonlinear hysteretic behavior represented by the Bouc–Wen internal variable is explained more clearly. In the proposed control framework, the Bouc–Wen model remains the mathematical representation of the MRE hysteresis, while the RBFNN does not replace this model. Instead, the RBFNN learns and compensates for the modeling uncertainties and nonlinear dynamics associated with the hysteretic restoring force represented by the internal variable (z(t)). Consequently, the Sliding Mode Controller receives an improved estimate of the nonlinear system dynamics, enabling more accurate control action, reduced chattering, and enhanced robustness against parameter variations and unmodeled hysteretic effects. The manuscript has been revised to clarify this interaction.

5 To better demonstrate the advantages of the proposed "Hybrid RBF-SMC", the current comparison mainly focuses on the Passive state and standard SMC.

**Response:**

We sincerely thank the reviewer for this valuable suggestion. We respectfully clarify that the present manuscript does not claim that the proposed Hybrid RBFNN–SMC controller outperforms a conventional Sliding Mode Controller. The primary objective of this work is to develop and demonstrate a hybrid adaptive control framework capable of maintaining effective vibration isolation under simultaneously varying excitation frequency and excitation amplitude, rather than to perform a comprehensive benchmarking study against multiple control algorithms.

The experimental characterization presented in this study establishes the current-dependent and amplitude-dependent dynamic properties of the fabricated MRE isolator. Based on these experimentally identified characteristics, a Hybrid RBF Neural Network–Sliding Mode Controller is developed to adaptively compensate for the nonlinear and hysteretic behavior of the isolator over the investigated operating range. Consequently, the simulation and experimental results are intended to demonstrate the controller's ability to maintain vibration reduction and stable operation under varying operating conditions, rather than to establish superiority over other control strategies.

For this reason, a detailed comparison with conventional Sliding Mode Control, constant-current control, fuzzy control, or other intelligent controllers has not been included in the present work. Such comparisons would require the implementation, parameter tuning, and validation of multiple controller architectures under identical operating conditions, which would substantially broaden the scope of the study.

The manuscript has been revised to clarify that the principal contribution of this work is the development and validation of an adaptive Hybrid RBFNN–SMC framework for frequency- and amplitude-varying excitation, and a comprehensive comparative study with conventional Sliding Mode Control and other advanced intelligent control methods has been identified as an important direction for future research.

6 The title highlights "Variable Frequency and Amplitude Excitations". While the single-frequency variations are well-presented, evaluating the system under more complex conditions would add significant value.

**Response:**

We sincerely thank the reviewer for this valuable suggestion. We agree that evaluating the proposed controller under more complex excitation conditions, such as multi-frequency, random, or broadband excitations, would provide additional insight into its robustness and practical applicability. However, the primary objective of the present study is to establish and validate the proposed Hybrid RBFNN–Sliding Mode Controller under controlled variable-frequency and variable-amplitude harmonic excitations, which represent the fundamental operating conditions for evaluating the adaptive behavior of the MRE isolator.

We acknowledge the importance of extending the validation to more realistic excitation environments and have added a statement in the Conclusion indicating that future work will focus on experimental validation under random, broadband, and multi-frequency excitations representative of real engineering applications.

7 To enhance readability, a dedicated Nomenclature list defining all Roman symbols, Greek symbols, and abbreviations would be a wonderful addition before the Introduction or in an Appendix.

**Response:**

To improve the readability of the manuscript, a dedicated **Nomenclature** section has been added before the Introduction. The section defines all major Roman symbols, Greek symbols, subscripts, and abbreviations used throughout the manuscript. This allows readers to quickly identify the notation employed in the mathematical modeling and controller design, thereby improving clarity and ease of understanding.

8 **Since intelligent control of smart materials is a rapidly evolving field, the authors are encouraged to include a few recent references from the past three years (2023-2026) regarding advanced control schemes for smart dampers/isolators to better reflect the latest state-of-the-art context.**

Ans. The Introduction has been revised to include several recent publications (2023–2026) covering advanced intelligent control strategies for smart dampers and magnetorheological elastomer-based vibration isolators. These newly added references include recent developments in adaptive control, neural-network-based control, fuzzy sliding mode control, hybrid intelligent controllers, and semi-active vibration control. The additional discussion provides a more comprehensive overview of the current state of the art and better positions the proposed Hybrid RBFNN–Sliding Mode Controller within the existing literature. The corresponding revisions have been incorporated in the Introduction (Control Strategy section), and the reference list has been updated accordingly.
