# Supplementary material for: Hybrid RBF neural network–based sliding mode control for a magnetorheological elastomer isolator under variable frequency and amplitude excitations
Source: PLoS One. 2026 Oct 9;21(10):e0360069. doi: 10.1371/journal.pone.0360069 (PMC13652330; doi:10.1371/journal.pone.0360069)
Supplement: Peer review history

# Peer review: Decision Letter 0

**DOI:** 10.1371/journal.pone.0360069.r001

**Authors:** Muhammad Usman

17 Jun 2026

Dear Dr. POOJARY,

Thank you for submitting your manuscript to PLOS ONE. After careful consideration, we feel that it has merit but does not fully meet PLOS ONE’s publication criteria as it currently stands. Therefore, we invite you to submit a revised version of the manuscript that addresses the points raised during the review process.

Please submit your revised manuscript by Aug 16 2026 11:59PM. If you will need more time than this to complete your revisions, please reply to this message or contact the journal office at plosone@plos.org. When you're ready to submit your revision, log on to https://www.editorialmanager.com/pone/ and select the 'Submissions Needing Revision' folder to locate your manuscript file.

- A letter that responds to each point raised by the academic editor and reviewer(s). You should upload this letter as a separate file labeled 'Response to Reviewers'.
- A marked-up copy of your manuscript that highlights changes made to the original version. You should upload this as a separate file labeled 'Revised Manuscript with Track Changes'.
- An unmarked version of your revised paper without tracked changes. You should upload this as a separate file labeled 'Manuscript'.

If you would like to make changes to your financial disclosure, please include your updated statement in your cover letter. Guidelines for resubmitting your figure files are available below the reviewer comments at the end of this letter.

If applicable, we recommend that you deposit your laboratory protocols in protocols.io to enhance the reproducibility of your results. Protocols.io assigns your protocol its own identifier (DOI) so that it can be cited independently in the future. For instructions see: https://journals.plos.org/plosone/s/submission-guidelines#loc-laboratory-protocols. Additionally, PLOS ONE offers an option for publishing peer-reviewed Lab Protocol articles, which describe protocols hosted on protocols.io. Read more information on sharing protocols at https://plos.org/protocols?utm_medium=editorial-email&utm_source=authorletters&utm_campaign=protocols.

As the corresponding author, your ORCID iD is verified in the submission system and will appear in the published article. PLOS supports the use of ORCID, and we encourage all coauthors to register for an ORCID iD and use it as well. Please encourage your coauthors to verify their ORCID iD within the submission system before final acceptance, as unverified ORCID iDs will not appear in the published article. *Only*  the individual author can complete the verification step; PLOS staff *cannot*  verify ORCID iDs on behalf of authors.

We look forward to receiving your revised manuscript.

Kind regards,

Muhammad Usman

Academic Editor

PLOS One

Journal Requirements:

When submitting your revision, we need you to address these additional requirements.

1. Please ensure that your manuscript meets PLOS ONE's style requirements, including those for file naming. The PLOS ONE style templates can be found at

https://journals.plos.org/plosone/s/file?id=wjVg/PLOSOne_formatting_sample_main_body.pdf and

https://journals.plos.org/plosone/s/file?id=ba62/PLOSOne_formatting_sample_title_authors_affiliations.pdf.

2. Please note that PLOS One has specific guidelines on code sharing for submissions in which author-generated code underpins the findings in the manuscript. In these cases, we expect all author-generated code to be made available without restrictions upon publication of the work. Please review our guidelines at https://journals.plos.org/plosone/s/materials-and-software-sharing#loc-sharing-code and ensure that your code is shared in a way that follows best practice and facilitates reproducibility and reuse.

3. We noted in your submission details that a portion of your manuscript may have been presented or published elsewhere. [Kiran Katari, Umanath R P, Ananda Hegde, and Gangadharan K V, “[Insert Full Title of Your Previous Paper],” [Journal Name], [Year], [Volume(Issue)], [Page numbers/Article ID].

These previously published results are included only for comparison and validation of the newly proposed Radial Basis Function Neural Network–based Sliding Mode (RBSM) control strategy developed in the present study. The primary contributions of this manuscript, including the hybrid controller formulation, mathematical modeling, Lyapunov stability analysis, neural network integration, and controller performance assessment, are original and have not been published or submitted elsewhere. Therefore, the present submission does not constitute duplicate publication. ] Please clarify whether this [conference proceeding or publication] was peer-reviewed and formally published. If this work was previously peer-reviewed and published, in the cover letter please provide the reason that this work does not constitute dual publication and should be included in the current manuscript.

4. In the online submission form you indicate that your data is not available for proprietary reasons and have provided a contact point for accessing this data. Please note that your current contact point is a co-author on this manuscript. According to our Data Policy, the contact point must not be an author on the manuscript and must be an institutional contact, ideally not an individual. Please revise your data statement to a non-author institutional point of contact, such as a data access or ethics committee, and send this to us via return email. Please also include contact information for the third party organization, and please include the full citation of where the data can be found.

5. PLOS requires an ORCID iD for the corresponding author in Editorial Manager on papers submitted after December 6th, 2016. Please ensure that you have an ORCID iD and that it is validated in Editorial Manager. To do this, go to ‘Update my Information’ (in the upper left-hand corner of the main menu), and click on the Fetch/Validate link next to the ORCID field. This will take you to the ORCID site and allow you to create a new iD or authenticate a pre-existing iD in Editorial Manager.

6. If the reviewer comments include a recommendation to cite specific previously published works, please review and evaluate these publications to determine whether they are relevant and should be cited. There is no requirement to cite these works unless the editor has indicated otherwise.

[Note: HTML markup is below. Please do not edit.]

Reviewers' comments:

Reviewer's Responses to Questions

**Comments to the Author**

1. Is the manuscript technically sound, and do the data support the conclusions?

Reviewer #1: Yes

Reviewer #2: Yes

Reviewer #3: Yes

**********

2. Has the statistical analysis been performed appropriately and rigorously?

Reviewer #1: Yes

Reviewer #2: Yes

Reviewer #3: Yes

**********

3. Have the authors made all data underlying the findings in their manuscript fully available?

The PLOS Data policy

Reviewer #1: Yes

Reviewer #2: Yes

Reviewer #3: Yes

**********

4. Is the manuscript presented in an intelligible fashion and written in standard English?

Reviewer #1: Yes

Reviewer #2: Yes

Reviewer #3: Yes

**********

Reviewer #1: This manuscript investigates the semi-active control of a magnetorheological elastomer (MRE) isolator under variable-frequency and variable-amplitude excitations. The topic has certain engineering significance, and the experimental characterization as well as the proposed RBFNN-SMC control strategy are generally clear. However, the manuscript still has several weaknesses regarding the consistency of reported results, the connection between experiments and simulations, the description of the controller design, and the sufficiency of performance validation. Therefore, I recommend major revision and reconsideration.

1. Inconsistency in key performance indicators.

The manuscript reports a maximum vibration reduction of approximately 65%–70% in the abstract, about 55.8% in the results section, and 55%–60% in the conclusion. Since these values represent the main performance claim of the study, the authors should unify the evaluation criteria and calculation basis, and ensure consistency across the abstract, results, and conclusion.

2. The link between experimental results and control simulations is not sufficiently clear.

The manuscript experimentally investigates the current-dependent and amplitude-dependent characteristics of the MRE isolator. However, the subsequent controller validation is mainly based on simulations. The authors should clarify how the experimentally obtained natural frequencies, stiffness, damping ratios, and frequency-shift characteristics are incorporated into the dynamic model and controller design.

3. Insufficient description of the RBF neural network.

The RBF neural network is used to approximate unknown nonlinearities, but important details such as the network architecture, input variables, number of hidden nodes, source of training data, and training error are not sufficiently provided. The authors should supplement these parameters and the training procedure to improve the reproducibility of the proposed method.

4. Further clarification of the sliding mode controller design is needed.

Although the manuscript provides the parameters of the sliding mode controller, the rationale for parameter selection, the stability proof, and the measures used to suppress chattering are not adequately explained. The authors should provide a clearer derivation of the control law, a more complete Lyapunov stability analysis, and the specific approach adopted to mitigate chattering.

5. The definition of the controller stopping frequency is not rigorous enough.

The manuscript proposes that the controller automatically switches off at certain frequencies, but the criterion for determining the stopping frequency is not clearly defined. The authors should provide a clear mathematical or engineering criterion, for example based on displacement transmissibility, control benefit, or frequency-response characteristics, to determine when the controller should be activated or deactivated.

6. The comparative validation is still insufficient.

The manuscript claims that the proposed controller outperforms the conventional sliding mode controller, but the comparison and evaluation metrics remain limited. The authors should provide a more systematic comparison with passive isolation, constant-current control, and conventional sliding mode control, using quantitative indicators such as peak response, RMS response, or displacement transmissibility.

Reviewer #2: The paper presents a “Study on the Implementation of Hybrid RBF Neural Network–Based Sliding Mode Control for a Magneto-rheological elastomer Isolator under Variable Frequency and Amplitude Excitations”. The work is interesting and the paper well written, but the following issues should be carefully addressed where (R) means “Recommendation. It does not compromise scientific rigour, but it compromises the rigour of communication and understanding.”:

1. (R) A general review by the authors is necessary, focusing on writing, correcting errors such as “charac-teristics”, ”uncertain-ties”, “model-ing” and others.

2. (R) There should be a more in-depth discussion of the practical and realistic application of the magnetorheological elastomer isolators.

Reviewer #3: The manuscript presents an interesting hybrid control strategy that combines a Radial Basis Function Neural Network (RBFNN) with a Sliding Mode Controller (SMC) for the semi-active vibration isolation of a magnetorheological elastomer (MRE) isolator. Given the inherent nonlinearities and hysteresis of MRE materials, exploring intelligent control combinations is highly meaningful for engineering applications. The manuscript is well-organized, and the preliminary results are encouraging. However, the manuscript could be significantly strengthened by refining the title, clarifying the core novelty, providing more details on controller feasibility, and expanding the baseline comparisons. A Major Revision is recommended to help the authors elevate the quality and impact of their work.

1 The current title is somewhat long and contains introductory phrasing (e.g., "A Study on the Implementation of..."), which can be streamlined to increase its academic readability and directness.

2 The current manuscript presents a solid engineering application, but the unique scientific contribution and specific advantages of this hybrid framework could be highlighted more prominently.

3 In standard sliding mode derivations, equivalent control terms often require high-order state derivatives. It would be helpful to clarify the practical execution of the RBFNN inputs.

4 The core challenge of MRE control lies in its nonlinear shear hysteresis loop, typically governed by the evolutionary internal variable z in the Bouc-Wen model. The text would benefit from a clearer explanation of how the RBFNN interacts with this specific hysteretic behavior.

5 To better demonstrate the advantages of the proposed "Hybrid RBF-SMC", the current comparison mainly focuses on the Passive state and standard SMC.

6 The title highlights "Variable Frequency and Amplitude Excitations". While the single-frequency variations are well-presented, evaluating the system under more complex conditions would add significant value.

7 To enhance readability, a dedicated Nomenclature list defining all Roman symbols, Greek symbols, and abbreviations would be a wonderful addition before the Introduction or in an Appendix.

8 Since intelligent control of smart materials is a rapidly evolving field, the authors are encouraged to include a few recent references from the past three years (2023-2026) regarding advanced control schemes for smart dampers/isolators to better reflect the latest state-of-the-art context.

**********

what does this mean?). If published, this will include your full peer review and any attached files.

If you choose “no”, your identity will remain anonymous but your review may still be made public.

**Do you want your identity to be public for this peer review?**  For information about this choice, including consent withdrawal, please see our Privacy Policy

Reviewer #1: No

Reviewer #2: No

Reviewer #3: No

**********

[NOTE: If reviewer comments were submitted as an attachment file, they will be attached to this email and accessible via the submission site. Please log into your account, locate the manuscript record, and check for the action link "View Attachments". If this link does not appear, there are no attachment files.]

To ensure your figures meet our technical requirements, please review our figure guidelines: https://journals.plos.org/plosone/s/figures

You may also use PLOS’s free figure tool, NAAS, to help you prepare publication quality figures: https://journals.plos.org/plosone/s/figures#loc-tools-for-figure-preparation.

NAAS will assess whether your figures meet our technical requirements by comparing each figure against our figure specifications.

Attachment Submitted filename: Comments-PONE-D-26-23860.doc

# Author comment: Author response to Decision Letter 1

**DOI:** 10.1371/journal.pone.0360069.r002

16 Jul 2026

Reviewer 1

1.The manuscript reports a maximum vibration reduction of approximately 65%–70% in the abstract, about 55.8% in the results section, and 55%–60% in the conclusion. Since these values represent the main performance claim of the study, the authors should unify the evaluation criteria and calculation basis, and ensure consistency across the abstract, results, and conclusion.

Response:

The manuscript has been carefully revised to ensure consistency in the reported vibration reduction values. The vibration reduction is now consistently evaluated using the percentage reduction in displacement transmissibility defined in Eq. (1). Accordingly, the Abstract, Results, and Conclusion have all been revised to report the same maximum vibration reduction of 55.8%, corresponding to the experimental conditions summarized in Table 3. These revisions ensure a uniform evaluation criterion and consistent reporting throughout the manuscript.

2. The link between experimental results and control simulations is not sufficiently clear. The manuscript experimentally investigates the current-dependent and amplitude-dependent characteristics of the MRE isolator. However, the subsequent controller validation is mainly based on simulations. The authors should clarify how the experimentally obtained natural frequencies, stiffness, damping ratios, and frequency-shift characteristics are incorporated into the dynamic model and controller design.

Response:

In the present study, the primary objective was to investigate the effectiveness of the proposed Radial Basis Sliding Mode (RBSM) control strategy in mitigating vibrations under deterministic harmonic excitation with varying frequencies. The excitation frequency range of 15–80 Hz was selected based on the operational conditions of the experimental vibration test setup used for evaluating the displacement transmissibility characteristics of the MRE isolator. The proposed controller is designed to adaptively regulate the control input according to the variation in excitation frequency, which allows the isolator to transition effectively between the active and passive operating states. The results presented in the manuscript demonstrate the capability of the proposed control strategy to reduce vibration transmissibility across the considered frequency band. The performance of the semi active MTRE isolator is assessed based on the simulation results.

However, a detailed investigation of the controller performance under needs to be carried out under random excitation inputs would require additional modelling of stochastic disturbances and extended simulation studies. Such an analysis, while valuable, is beyond the scope of the current manuscript. This would be addressed in the separate communication.

As highlighted by the reviewer, in the revised manuscript, the relationship between the experimental characterization and the subsequent controller simulations should be explained more clearly. Additionally, manuscript has been revised to clarify that the experimentally obtained dynamic characteristics of the MRE isolator, including the natural frequency, equivalent stiffness, damping ratio, and current-dependent frequency-shift behavior, were used to parameterize and validate the dynamic model employed in the controller simulations. The Bouc–Wen model parameters and the equivalent dynamic properties were selected to reproduce the experimentally observed behavior of the MRE isolator under different excitation amplitudes and coil current levels. Consequently, the simulation results are based on a dynamic model calibrated using the experimentally measured characteristics rather than purely theoretical assumptions.

3. Insufficient description of the RBF neural network.

The RBF neural network is used to approximate unknown nonlinearities, but important details such as the network architecture, input variables, number of hidden nodes, source of training data, and training error are not sufficiently provided. The authors should supplement these parameters and the training procedure to improve the reproducibility of the proposed method.

Response:

The additional implementation details of the RBF Neural Network improve the reproducibility of the proposed controller. Accordingly, the manuscript has been revised to provide a more detailed description of the RBFNN architecture, including the network inputs, hidden layer configuration, radial basis activation function, network output, and adaptive weight update mechanism. Furthermore, the manuscript now clarifies that the RBFNN operates as an online adaptive approximator within the control loop, where the network weights are updated according to the Lyapunov-based adaptation law using measured system states, rather than through offline supervised training. These additions clarify the implementation procedure and improve the reproducibility of the proposed method.

4. Further clarification of the sliding mode controller design is needed.

Although the manuscript provides the parameters of the sliding mode controller, the rationale for parameter selection, the stability proof, and the measures used to suppress chattering are not adequately explained. The authors should provide a clearer derivation of the control law, a more complete Lyapunov stability analysis, and the specific approach adopted to mitigate chattering.

Response:

The design methodology of the proposed Sliding Mode Controller explained in greater detail. Accordingly, the manuscript has been revised to clarify the rationale for selecting the sliding surface and controller parameters, the derivation of the control law, and the Lyapunov-based stability analysis. In addition, the manuscript now explains how the adaptive RBF Neural Network estimates the unknown nonlinear dynamics, thereby reducing the discontinuous switching action required by the Sliding Mode Controller and mitigating control chattering. These additions improve the transparency and reproducibility of the proposed control strategy.

5. The definition of the controller stopping frequency is not rigorous enough.

The manuscript proposes that the controller automatically switches off at certain frequencies, but the criterion for determining the stopping frequency is not clearly defined. The authors should provide a clear mathematical or engineering criterion, for example based on displacement transmissibility, control benefit, or frequency-response characteristics, to determine when the controller should be activated or deactivated.

Response:

The controller stopping frequency is a unique term coined by the author to distinguish between the active and the passive switch over state of the controller. Stopping frequency is a limiting frequency which could eliminate the risk of instability of the system due to continued active state. Without knowing the stopping frequency, the controller will not have the clue about the when the isolator should be passive to prevent the system reaching the resonance of the active state. Since the cut off frequency is unique for a specific isolator, developing a generalized mathematical approach would not be feasible. However as stated by the reviewer, the controller stopping frequency is defined using a clearer engineering criterion. Accordingly, the manuscript has been revised to define the stopping frequency based on the displacement transmissibility response of the MRE isolator. In the revised manuscript, the controller is activated when the controlled state provides a measurable reduction in displacement transmissibility compared with the passive state. Conversely, the controller is deactivated when the improvement in transmissibility becomes negligible or when passive isolation is already sufficient. This criterion provides a clear basis for determining the controller activation and deactivation regions.

6. The comparative validation is still insufficient.

The manuscript claims that the proposed controller outperforms the conventional sliding mode controller, but the comparison and evaluation metrics remain limited. The authors should provide a more systematic comparison with passive isolation, constant-current control, and conventional sliding mode control, using quantitative indicators such as peak response, RMS response, or displacement transmissibility.

Response:

We sincerely thank the reviewer for this valuable suggestion. We respectfully clarify that the present manuscript does not claim that the proposed Hybrid RBFNN–SMC controller outperforms a conventional Sliding Mode Controller. The primary objective of this work is to develop and demonstrate a hybrid adaptive control framework capable of maintaining effective vibration isolation under simultaneously varying excitation frequency and excitation amplitude, rather than to perform a comprehensive benchmarking study against multiple control algorithms.

The experimental characterization presented in this study establishes the current-dependent and amplitude-dependent dynamic properties of the fabricated MRE isolator. Based on these experimentally identified characteristics, a Hybrid RBF Neural Network–Sliding Mode Controller is developed to adaptively compensate for the nonlinear and hysteretic behavior of the isolator over the investigated operating range. Consequently, the simulation and experimental results are intended to demonstrate the controller's ability to maintain vibration reduction and stable operation under varying operating conditions, rather than to establish superiority over other control strategies.

For this reason, a detailed comparison with conventional Sliding Mode Control, constant-current control, fuzzy control, or other intelligent controllers has not been included in the present work. Such comparisons would require the implementation, parameter tuning, and validation of multiple controller architectures under identical operating conditions, which would substantially broaden the scope of the study.

The manuscript has been revised to clarify that the principal contribution of this work is the development and validation of an adaptive Hybrid RBFNN–SMC framework for frequency- and amplitude-varying excitation, and a comprehensive comparative study with conventional Sliding Mode Control and other advanced intelligent control methods has been identified as an important direction for future research.

Reviewer 2

1. (R) A general review by the authors is necessary, focusing on writing, correcting errors such as “charac-teristics”, ”uncertain-ties”, “model-ing” and others.

Response:

The entire manuscript has been carefully proofread and revised to improve the quality of the writing. Typographical errors, word breaks, grammatical inconsistencies, and formatting issues—including examples such as "charac-teristics", "uncertain-ties", and "model-ing"—have been corrected throughout the manuscript. In addition, the text has been edited to improve readability, consistency, and overall language quality.

2. (R) There should be a more in-depth discussion of the practical and realistic application of the magnetorheological elastomer isolators.

As suggested by the reviewer, the introduction section is modified by adding additional information on the practical and realistic applications of MRE

Reviewer 3

1 The current title is somewhat long and contains introductory phrasing (e.g., "A Study on the Implementation of..."), which can be streamlined to increase its academic readability and directness.

Response:

We sincerely thank the reviewer for this valuable suggestion. We agree that the original title was unnecessarily lengthy and contained introductory phrasing that reduced its directness. Accordingly, the title has been revised to improve its academic readability while preserving the technical scope of the study.

Revised Title:

Hybrid RBF Neural Network-Based Sliding Mode Control for a Magnetorheological Elastomer Isolator under Variable Frequency and Amplitude Excitations

2 The current manuscript presents a solid engineering application, but the unique scientific contribution and specific advantages of this hybrid framework could be highlighted more prominently.

Response:

Accordingly, the Introduction has been revised to explicitly highlight the novelty of the proposed approach. Unlike conventional Sliding Mode Control, the proposed framework integrates an adaptive RBF Neural Network with a physics-based Bouc–Wen hysteresis model to compensate for nonlinear modeling uncertainties while preserving the robustness of Sliding Mode Control. Furthermore, the controller is specifically designed for MRE isolators operating under simultaneously varying excitation frequency and amplitude, allowing adaptive compensation of hysteretic nonlinearities without requiring an exact system model. These scientific contributions and their advantages over conventional control strategies have been clarified in both the Introduction and the Conclusion.

3 In standard sliding mode derivations, equivalent control terms often require high-order state derivatives. It would be helpful to clarify the practical execution of the RBFNN inputs.

Response:

The practical implementation of the RBF Neural Network inputs is explained more clearly. In the proposed controller, the RBFNN uses only the tracking error and its first derivative as input variables. These quantities are directly obtained from the measured displacement response and the desired reference trajectory, and therefore no higher-order state derivatives are required for online implementation. The RBFNN adaptively estimates the unknown nonlinear dynamics based on these measurable signals, while the Sliding Mode Controller utilizes this estimate to generate the control action. Additional clarification has been added to the manuscript to explain the practical realization of the RBFNN inputs.

4 The core challenge of MRE control lies in its nonlinear shear hysteresis loop, typically governed by the evolutionary internal variable z in the Bouc-Wen model. The text would benefit from a clearer explanation of how the RBFNN interacts with this specific hysteretic behavior.

Response:

The interaction between the proposed RBF Neural Network and the nonlinear hysteretic behavior represented by the Bouc–Wen internal variable is explained more clearly. In the proposed control framework, the Bouc–Wen model remains the mathematical representation of the MRE hysteresis, while the RBFNN does not replace this model. Instead, the RBFNN learns and compensates for the modeling uncertainties and nonlinear dynamics associated with the hysteretic restoring force represented by the internal variable (z(t)). Consequently, the Sliding Mode Controller receives an improved estimate of the nonlinear system dynamics, enabling more accurate control action, reduced chattering, and enhanced robustness against parameter variations and unmodeled hysteretic effects. The manuscript has been revised to clarify this interaction.

5 To better demonstrate the advantages of the proposed "Hybrid RBF-SMC", the current comparison mainly focuses on the Passive state and standard SMC.

Response:

We sincerely thank the reviewer for this valuable suggestion. We respectfully clarify that the present manuscript does not claim that the proposed Hybrid RBFNN–SMC controller outperforms a conventional Sliding Mode Controller. The primary objective of this work is to develop and demonstrate a hybrid adaptive control framework capable of maintaining effective vibration isolation under simultaneously varying excitation frequency and excitation amplitude, rather than to perform a comprehensive benchmarking study against multiple control algorithms.

The experimental characterization presented in this study establishes the current-dependent and amplitude-dependent dynamic properties of the fabricated MRE isolator. Based on these experimentally identified characteristics, a Hybrid RBF Neural Network–Sliding Mode Controller is developed to adaptively compensate for the nonlinear and hysteretic behavior of the isolator over the investigated operating range. Consequently, the simulation and experimental results are intended to demonstrate the controller's ability to maintain vibration reduction and stable operation under varying operating conditions, rather than to establish superiority over other control strategies.

For this reason, a detailed comparison with conventional Sliding Mode Control, constant-current control, fuzzy control, or other intelligent controllers has not been included in the present work. Such comparisons would require the implementation, parameter tuning, and validation of multiple controller architectures under identical operating conditions, which would substantially broaden the scope of the study.

The manuscript has been revised to clarify that the principal contribution of this work is the development and validation of an adaptive Hybrid RBFNN–SMC framework for frequency- and amplitude-varying excitation, and a comprehensive comparative study with conventional Sliding Mode Control and other advanced intelligent control methods has been identified as an important direction for future research.

6 The title highlights "Variable Frequency and Amplitude Excitations". While the single-frequency variations are well-presented, evaluating the system under more complex conditions would add significant value.

Response:

We sincerely thank the reviewer for this valuable suggestion. We agree that evaluating the proposed controller under more complex excitation conditions, such as multi-frequency, random, or broadband excitations, would provide additional insight into its robustness and practical applicability. However, the primary objective of the present study is to establish and validate the proposed Hybrid RBFNN–Sliding Mode Controller under controlled variable-frequency and variable-amplitude harmonic excitations, which represent the fundamental operating conditions for evaluating the adaptive behavior of the MRE isolator.

We acknowledge the importance of extending the validation to more realistic excitation environments and have added a statement in the Conclusion indicating that future work will focus on experimental validation under random, broadband, and multi-frequency excitations representative of real engineering applications.

7 To enhance readability, a dedicated Nomenclature list defining all Roman symbols, Greek symbols, and abbreviations would be a wonderful addition before the Introduction or in an Appendix.

Response:

To improve the readability of the manuscript, a dedicated Nomenclature section has been added before the Introduction. The section defines all major Roman symbols, Greek symbols, subscripts, and abbreviations used throughout the manuscript. This allows readers to quickly identify the notation employed in the mathematical modeling and controller design, thereby improving clarity and ease of understanding.

8 Since intelligent control of smart materials is a rapidly evolving field, the authors are encouraged to include a few recent references from the past three years (2023-2026) regarding advanced control schemes for smart dampers/isolators to better reflect the latest state-of-the-art context.

Ans. The Introduction has been revised to include several recent publications (2023–2026) covering advanced intelligent control strategies for smart dampers and magnetorheological elastomer-based vibration isolators. These newly added references include recent developments in adaptive control, neural-network-based control, fuzzy sliding mode control, hybrid intelligent controllers, and semi-active vibration control. The additional discussion provides a more comprehensive overview of the current state of the art and better positions the proposed Hybrid RBFNN–Sliding Mode Controller within the existing literature. The corresponding revisions have been incorporated in the Introduction (Control Strategy section), and the reference list has been updated accordingly.

Attachment Submitted filename: Comments-PONE-D-26-23860_ reviewed.doc

# Peer review: Decision Letter 1

**DOI:** 10.1371/journal.pone.0360069.r003

**Authors:** Muhammad Usman

23 Sep 2026

Hybrid RBF Neural Network–Based Sliding Mode Control for a Magnetorheological Elastomer Isolator under Variable Frequency and Amplitude Excitations

PONE-D-26-23860R1

Dear Dr. POOJARY,

We’re pleased to inform you that your manuscript has been judged scientifically suitable for publication and will be formally accepted for publication once it meets all outstanding technical requirements.

Within one week, you’ll receive an e-mail detailing the required amendments. When these have been addressed, you’ll receive a formal acceptance letter and your manuscript will be scheduled for publication.

An invoice will be generated when your article is formally accepted. Please note, if your institution has a publishing partnership with PLOS and your article meets the relevant criteria, all or part of your publication costs will be covered. Please make sure your user information is up-to-date by logging into Editorial Manager at Editorial Manager® and clicking the ‘Update My Information' link at the top of the page. For questions related to billing, please contact billing support.

If your institution or institutions have a press office, please notify them about your upcoming paper to help maximize its impact. If they’ll be preparing press materials, please inform our press team as soon as possible -- no later than 48 hours after receiving the formal acceptance. Your manuscript will remain under strict press embargo until 2 pm Eastern Time on the date of publication. For more information, please contact onepress@plos.org.

Kind regards,

Muhammad Usman

Academic Editor

PLOS One

Additional Editor Comments (optional):

Reviewers' comments:

Reviewer's Responses to Questions

**Comments to the Author**

Reviewer #3: All comments have been addressed

**********

2. Is the manuscript technically sound, and do the data support the conclusions?

Reviewer #3: Yes

**********

3. Has the statistical analysis been performed appropriately and rigorously?

Reviewer #3: Yes

**********

4. Have the authors made all data underlying the findings in their manuscript fully available?

The PLOS Data policy

Reviewer #3: Yes

**********

5. Is the manuscript presented in an intelligible fashion and written in standard English?

Reviewer #3: Yes

**********

Reviewer #3: The manuscript presents an interesting hybrid control strategy that combines a Radial Basis Function Neural Network (RBFNN) with a Sliding Mode Controller (SMC) for the semi-active vibration isolation of a magnetorheological elastomer (MRE) isolator. After revision, this manuscript can be accepted in the current form.

**********

what does this mean?). If published, this will include your full peer review and any attached files.

If you choose “no”, your identity will remain anonymous but your review may still be made public.

**Do you want your identity to be public for this peer review?**  For information about this choice, including consent withdrawal, please see our Privacy Policy

Reviewer #3: No

**********

# Editor report: Acceptance letter

**DOI:** 10.1371/journal.pone.0360069.r004

**Authors:** Muhammad Usman

PONE-D-26-23860R1

PLOS One

Dear Dr. POOJARY,

I'm pleased to inform you that your manuscript has been deemed suitable for publication in PLOS One. Congratulations! Your manuscript is now being handed over to our production team.

At this stage, our production department will prepare your paper for publication. This includes ensuring the following:

* All references, tables, and figures are properly cited

* All relevant supporting information is included in the manuscript submission,

* There are no issues that prevent the paper from being properly typeset

You will receive further instructions from the production team, including instructions on how to review your proof when it is ready. Please keep in mind that we are working through a large volume of accepted articles, so please give us a few days to review your paper and let you know the next and final steps.

Lastly, if your institution or institutions have a press office, please let them know about your upcoming paper now to help maximize its impact. If they'll be preparing press materials, please inform our press team within the next 48 hours. Your manuscript will remain under strict press embargo until 2 pm Eastern Time on the date of publication. For more information, please contact onepress@plos.org.

You will receive an invoice from PLOS for your publication fee after your manuscript has reached the completed accept phase. If you receive an email requesting payment before acceptance or for any other service, this may be a phishing scheme. Learn how to identify phishing emails and protect your accounts at https://explore.plos.org/phishing.

If we can help with anything else, please email us at customercare@plos.org.

Thank you for submitting your work to PLOS One and supporting open access.

Kind regards,

PLOS One Editorial Office Staff

on behalf of

Dr. Muhammad Usman

Academic Editor

PLOS One
